# The processing of the Dutch masculine generic *zijn* ‘his’ across stereotype contexts: An eye-tracking study

**DOI:** 10.1371/journal.pone.0205903

**Published:** 2018-10-18

**Authors:** Theresa Redl, Anita Eerland, Ted J. M. Sanders

**Affiliations:** 1 Centre for Language Studies, Radboud University, Nijmegen, The Netherlands; 2 Max Planck Institute for Psycholinguistics, Nijmegen, The Netherlands; 3 Department of Languages, Literature and Communication, Utrecht Institute of Linguistics, Utrecht University, Utrecht, The Netherlands; Leiden University, NETHERLANDS

## Abstract

Language users often infer a person’s gender when it is not explicitly mentioned. This information is included in the mental model of the described situation, giving rise to expectations regarding the continuation of the discourse. Such gender inferences can be based on two types of information: gender stereotypes (e.g., nurses are female) and masculine generics, which are grammatically masculine word forms that are used to refer to all genders in certain contexts (e.g., *To each* ***his*** *own*). In this eye-tracking experiment (*N* = 82), which is the first to systematically investigate the online processing of masculine generic pronouns, we tested whether the frequently used Dutch masculine generic *zijn* ‘his’ leads to a male bias. In addition, we tested the effect of context by introducing male, female, and neutral stereotypes. We found no evidence for the hypothesis that the generically-intended masculine pronoun *zijn* ‘his’ results in a male bias. However, we found an effect of stereotype context. After introducing a female stereotype, reading about a man led to an increase in processing time. However, the reverse did not hold, which parallels the finding in social psychology that men are penalized more for gender-nonconforming behavior. This suggests that language processing is not only affected by the strength of stereotype contexts; the associated disapproval of violating these gender stereotypes affects language processing, too.

## Introduction

Masculine forms are often used when reference to people in general is made. This phenomenon is, for example, apparent in the proverb *To each his own*, which applies to men and women alike, but yet features the masculine pronoun *his*. Many of the world’s languages exhibit this phenomenon [1,2], one of them being Dutch [3]. Consider the following example, a headline taken from a column in the Dutch quality newspaper *De Volkskrant* [4] further illustrating this practice:

(1) *Elke postbezorger zal zich moeten afvragen wat hij kan doen om als geheel sterker te staan*.
  ‘Every postal worker will have to think about what he can do for all postal workers to gain a better standing as a group.’

Dutch natives will likely assume that the author of this piece intended to refer to all postal workers, not only to the male ones. In other words, *hij* ‘he’ is used *generically*, as referring to men as well as women, despite carrying masculine grammatical gender. Such word forms, which carry masculine grammatical gender, but are used generically, are usually referred to as generic masculines [5–7] or masculine generics [8–10]. They are more precisely defined as masculine forms that are used to refer to people of unknown or unspecified gender or to groups of mixed gender [9,11,12].

Crucially, masculine terms that can serve as masculine generics are ambiguous between two readings: A generic reading and a male-specific reading. For example, the headline above allows for the generic reading, including all postal workers regardless of their gender. Alternatively, a male-specific reading, for which it is only the male postal workers who ought to organize themselves, is also available. Context may resolve the ambiguity of masculine generics, but completely straightforward cases are rare.

In the 1970s, criticism of masculine generics and their ambiguity grew louder, first in the English-speaking countries [13,14] and later spreading to other countries such as Germany [15] and the Netherlands [16]. The claim that masculine generics can refer to men and women alike was challenged. Opponents of masculine generics, for example Silveira [17], suggested that the ambiguity of the masculine generic is resolved to women’s disadvantage by being interpreted as male-specific, thereby rendering women linguistically invisible. Early research on masculine generics such as *he* and *his* in English soon suggested that, although intended as generic, the use of masculine generics indeed results in a male bias. For example, Moulton et al. [14] found that when a sentence about a hypothetical person featured the masculine generic pronoun *his* (e.g., *In a large coeducational institution the average student will feel isolated in his introductory courses*), this hypothetical person was thought of as male rather than female. A comparable male bias by English masculine generic pronouns was found by other researchers between the 1970s and 1990s [9,18–21] as well as more recently [22]. However, these studies made use of rather explicit methods as a means of tapping into the hypothesized male bias, such as writing a story about a character or describing the images that came to mind when reading. For example, in their aforementioned experiment, Moulton et al. [14] provided participants with the description of a hypothetical person fitting either of two themes (i.e., *being a student* or *being concerned with looks*), and the masculine generic pronoun *his* was used to describe this person. Moulton et al. [14] asked their participants to write a story about a fictitious person fitting these themes. The gender which participants chose for their character in the story then served as the dependent variable. Thus, the authors gave participants ample time to decide on their choice of gender for their character by employing this design. Put differently, participants were given time to ponder whether the masculine generic was intended as generic or male-specific. As a result, they might have chosen to write about a male character more often as this is the safe choice; writing about a male character fits with the male-specific as well as with the generic reading of the pronoun. A female character, however, only makes sense in the context of a generic reading of the pronoun. In sum, many of these early studies found that generically-intended masculine pronouns lead to a male bias, but they did so using rather explicit research methods, which reveal little about the actual *processing* of masculine generics. Moreover, one study failed to find an effect of a male bias induced by generic *he* altogether [23]. Hence, the question remains if generic pronouns lead to a male bias in online processing.

In the last 20 years, the research focus regarding masculine generics has shifted from English pronouns towards other Indo-European languages and so-called role nouns. Role nouns are generally defined as “any names that incorporate features used to describe a person or a group of people, such as hobbies (e.g., soccer fan) or occupations (e.g., dentists, actors, or students)” [24]. In languages such as French and German, role nouns are marked for gender and the masculine form is used as the default (e.g., German *der durchschnittliche Student*, ‘the average student, *masculine*’, or French *un professeur sévère*, ‘a strict teacher, *masculine*’). It has been repeatedly shown that generically-intended masculine role nouns are interpreted as referring to men rather than women. Contrary to previous research on masculine generic pronouns, the male bias of role nouns has been observed using various online methods such as self-paced reading [25], eye-tracking [26,27], the sentence evaluation paradigm [12,28] and EEG [29].

With one exception [29], all listed experiments investigated the potential male bias of role nouns as masculine generics across different stereotype contexts, thus combining the research into these two types of gender cues. Past research has shown that gender stereotypes (e.g., nurses are typically female) are a powerful trigger of gender inferences, in the presence as well as in the absence of unambiguous gender cues [30–33]. For example, Carreiras et al. [30] showed that the referent in sentences such as *The electrician examined the light fitting* is thought of as male rather than female, although no explicit reference to the subject’s gender is made. Thus, stereotypes can be used to enrich the mental representation of a referent [34]. Put differently, a stereotype can trigger a gender inference when a referent’s gender is not explicitly stated. Previous research on role nouns as masculine generics suggests that they, too, can give rise to gender inferences. In this latter case, the masculine grammatical gender of a masculine generic is erroneously interpreted as an indication of the referent(s)’ gender. Many researchers combined these two different types of gender cues, one grammatical in nature, the other stemming from world knowledge. This allowed researchers to test whether masculine generics cause a male bias at all, and whether they do so in the context of other gender information. This approach takes into account that context strongly affects the interpretation of ambiguous lexical items [35], leading to a better understanding of how masculine generics are processed and how gender inferences are made.

To summarize, research into the processing of masculine generics has been largely restricted to role nouns until now. We do not know whether masculine generic pronouns give rise to gender inferences in a similar online, automatic, and elaborative fashion as stereotypes and masculine generic role nouns, since past research into masculine generic pronouns has made use of arguably explicit and often offline methods.

To fill this gap, we extended the line of processing research recently applied to stereotypical role nouns to the Dutch masculine generic *zijn* ‘his’ and conducted an eye-tracking experiment as a means of tapping into language processing directly. By presenting *zijn* in female, male and neutral stereotype contexts (e.g., *Iedereen was zijn tanden aan het poetsen* ‘Everyone was brushing his teeth’), we were able to test whether this masculine generic pronoun leads to a male bias in processing, and whether the hypothesized male bias persists across contexts.

There are reasons to believe that masculine generic pronouns work differently from masculine generic role nouns. When a masculine pronoun is used as a generic such as Dutch *zijn* ‘his’, it is always that very same token that is used in generic contexts. In the case of generically-intended role nouns, an arguably infinite number of tokens is used. Their pattern is of course the same: A grammatically masculine noun is used to refer to people in general, but other than with pronouns there is a vast list of tokens. Being lexical in nature, role nouns give rise to frequency effects. These in turn might make it hard to generalize experimental findings based on a subset of role nouns to the whole set of role nouns in a language if frequency effects are not controlled for. More specifically, if the grammatically feminine form of a role noun is very frequent, we expect the grammatically masculine counterpart to be interpreted as male-specific and having a lower generic potential than would be the case for a masculine role noun of which the feminine counterpart is highly infrequent. Thus, if a masculine role noun is used generically a lot—or even to refer to female individuals—we expect its generic potential to be higher. De Backer and De Cuypere [36] indeed found evidence that the relative frequency of the masculine and feminine form of a role noun affects whether the masculine form is easily interpreted as generic. This suggests that not all masculine generic role nouns, not even within the same language, work the same way. This might also partly explain why there is no consensus yet, as to whether the grammatical gender of generic role nouns overrules stereotype context or not. For example, Gygax et al. [12] and Garnham et al. [28], on the one hand, found evidence for a male bias across all contexts. Irmen and Roßberg [25], on the other hand, found that the two types of gender cues interacted and that context may weaken the masculine generic’s male bias. This confounding factor of varied relative frequency per role noun is not an issue when it comes to the processing of masculine generic pronouns. Take the Dutch possessive pronoun *zijn* ‘his’ as a masculine generic: There is only one token, and this token is presumably more frequent than most role noun tokens. Of course, frequency might still affect the processing of *zijn*, too: The relative frequency of generic *zijn* and male-specific *zijn*, and possibly the frequency of the pronoun’s feminine counterpart *haar* ‘her’ might affect the generic potential of the pronoun. If this is indeed the case, then this effect of relative frequency is held constant and therefore controlled for within an experiment. Therefore, the results and the conclusions are not affected by the choice of tokens, as might be the case for role nouns. There are several ways in which relative frequency might affect the reading of a pronoun. A generically-intended pronoun such as *zijn* might lead to a strong male bias if the generic reading of the pronoun is only weakly represented. If, however, the generic reading of *zijn* is relatively frequent and more strongly represented, this pronoun might exhibit a stronger generic potential than has been found for role nouns. Finally, it is also possible that a masculine generic pronoun can never be interpreted as truly generic, even if the generic reading is strongly represented.

Another difference between role nouns and the possessive pronoun specifically lies in the salience of the two. To our knowledge, all experiments on role nouns have made use of stimuli which introduced the role noun in subject position. In these stimuli, the role noun further usually constituted the first mention of the referent(s) denoted by the role noun. Both these things are different for the possessive pronoun, at least in the linguistic structure we chose for our stimulus design. First of all, we used the possessive pronoun anaphorically. Thus, the referent is previously introduced and then referred back to by means of the possessive pronoun. Furthermore, the possessive pronoun is part of a larger noun phrase, as is often the case with possessive pronouns, and this noun phrase occurs in object position. These are all factors that might lower the salience of *zijn* ‘his’ and might therefore decrease the impact of the grammatical gender on the mental representation of the referent, or in other words boost the generic potential of the pronoun.

In sum, the online processing of masculine generic pronouns has not previously been thoroughly investigated and will be the focus of the present study. We cannot make clear predictions based on research into role nouns, but there is reason to believe that masculine generic pronouns might not work the same. This study is also a first for research into the online processing of any type of masculine generic in Dutch. While criticism of masculine generics in the Dutch language was voiced very early on [16], experimental studies on the online processing of Dutch masculine generics are still non-existent—despite masculine generics still being commonly used in Dutch. The few empirical offline studies on Dutch masculine generics which exist do suggest that they may induce a male bias. A questionnaire by De Backer and De Cuypere [36] suggests that generically-intended Dutch role nouns are often not interpreted as generic. In addition, two psychological studies by Vervecken and Hannover [37] and Vervecken et al. [38] have shown that Dutch masculine role nouns negatively affect the mental accessibility of female jobholders and children’s self-efficacy. Again, it is hard to base concrete predictions regarding our eye-tracking reading experiment featuring the Dutch masculine generic *zijn* ‘his’ across stereotype contexts on role noun research, particularly because the grammatical gender of role nouns has been found to overwrite stereotype context by some [12,28], but the two factors have been found to interact by others [25]. More specifically, Irmen and Roßberg [25] found that the combination of a masculine role noun and a female stereotype prepares readers equally well for a female and a male referent. Given the evidence that stereotype context can interact with grammatical gender regarding role nouns [25], and the less salient nature of *zijn* compared to role nouns, we predicted that the masculine generic *zijn* results in a male bias in neutral and male contexts only. Thus, if the context is neutral and *zijn* thus constitutes the only gender cue, we expected a male bias to emerge. We expected similar results for male stereotype contexts, as both *zijn* and the context suggest a male referent. In female stereotype contexts, however, the two gender cues make contrary predictions and we expected the male bias of the masculine generic *zijn* to be attenuated or even cancelled out.

## Materials and methods

### Participants

We tested a total of 92 participants (42 male) between the ages of 18 and 51 (*M* = 22.8, *SD* = 4.6), who gave written consent to participating in the experiment. We declare that at present and at the time of the study, the Utrecht Institute of Linguistics, where the research was conducted, endorses the WMA Declaration of Helsinki, as well as The Netherlands Code of Conduct for Scientific Practice by the Association of Universities in the Netherlands (VSNU). Participants were recruited largely through the participant database of the Utrecht Institute of Linguistics Lab at Utrecht University, but separate calls for male participants were placed online. The first language of all 92 participants was Dutch, with five participants being multilingual. A total of 88 participants were students, three were working and one was a stay-at-home parent. All participants had normal or corrected-to-normal vision. They were paid €5.- for their participation. The experiment took approximately 25 minutes.

Two exclusion criteria applied. First, participants were required to answer more than 75% of the comprehension questions correctly in order for their data to be considered in the analysis. This was done to make sure that participants actually read the sentences for comprehension. Second, we excluded participants who correctly guessed the purpose of the experiment on the exit questionnaire. This was defined as either (a) describing a mismatch between the gender stereotype and the gender of a mentioned individual or (b) describing a mismatch between the masculine gender suggested by the masculine generic *zijn* and the gender of a mentioned individual, or both.

### Materials

Each participant was presented with 96 Dutch sentence pairs: 48 experimental stimuli, 12 control items and 36 fillers (see S1 Appendix for a full list of experimental stimuli and control items). The experimental stimuli all conformed to the same pattern, with a group of people being introduced in the first sentence and an individual of this group being referred to in the second sentence. The following example stimulus illustrates this pattern:

(2) *Iedereen was zijn tanden aan het poetsen*. *Zo was ook Daphne/Robert zich aan het klaarmaken om naar bed te gaan*.
  ‘Everyone was brushing his teeth. Daphne/Robert, too, was getting ready to go to bed.’

In the first sentence of the experimental stimuli, a group was introduced by means of the quantifier *iedereen* ‘everyone’. All members of this group were engaging in a particular activity (e.g., *brushing teeth*). These activities were always expressed by means of the possessive pronoun and masculine generic *zijn* ‘his’ introducing a direct object, followed by a progressive verb form. These activities were further intended to introduce a gender stereotype. These stereotypes were either stereotypically female (e.g., *yogaoefeningen doen* ‘doing yoga exercises’), male (e.g., *voetbaltrucs oefenen* ‘practicing soccer tricks’) or neutral (e.g., *tanden poetsen* ‘brushing teeth’) according to a pre-test (see below for details on this pre-test). In the second sentence of the experimental stimuli, reference was made to either a male or a female individual by means of a proper name. These names were carefully selected from the *Nederlandse Voornamenbank* ‘Dutch first name database’ by the Meertens Institute [39]. We selected 30 male and 30 female proper names (48 for the experimental stimuli, twelve for the controls) from the annual list of the 60 most popular names in the Netherlands from 1990 through 2009. All names count two syllables and are between four and six characters long. We further only chose names which could be identified as either unambiguously male or female, as agreed upon by three native speakers. These three native speakers further also evaluated all sentence pairs regarding the likelihood that the mentioned individual would be interpreted as being part of the group mentioned in the first sentence. This was the case for all items.

Of the 48 experimental items, 16 featured male, female and neutral activities, respectively. Within each stereotype category, half the stimuli (i.e., eight sentence pairs) featured a female name, while the other half featured a male name. An overview of the design can be found in Table 1. Two lists were created to make sure that each sentence pair occurred with both a female and a male continuation and participants were pseudo-randomly assigned to either list.

**Table 1:** Design overview with number of stimuli.

		*Stereotype*			
		Female	Male	Neutral	*Total*
*Continuation*	Female	8	8	8	24
	Male	8	8	8	24
	*Total*	16	16	16	48

The purpose of the twelve control items was to assure that any observed effects were indeed due to the experimental manipulation, i.e. the occurrence of the masculine generic *zijn* and/or the biasing male and female stereotype context and not due to a more general male bias as has been previously suggested by some researchers [17,25,26]. Therefore, the controls differed from the experimental items in two ways. First, the masculine generic *zijn* was omitted. Second, only neutral (and therefore non-biasing) activities were used. The following example illustrates the design of the control items:

(3) *Iedereen was een treinkaartje aan het kopen*. *Zo was ook Amber bij het loket in de rij gaan staan*.
  ‘Everyone was buying a train ticket. Amber, too, had gotten in line at the counter.’

As with the experimental items, half of the controls featured a male or female proper name, respectively. Again, this was counterbalanced across lists.

The 36 filler items were designed to mask the experiment’s purpose and featured neither the masculine generic *zijn* nor female or male individuals:

(4) *Iedereen was de toets aan het maken*. *Ze hadden er bijna twee uur de tijd voor*.
  ‘Everyone was taking the test. They had almost two hours.’

After half of all 96 sentence pairs, evenly distributed over experimental, control and filler items, a statement about the content was displayed. For example, participants had to respond to the statement in (5) after reading the sentence pair in (4).

(5) *De toets duurde een half uur*.
  ‘The test took half an hour.’

These statements had to be judged as correct or incorrect. The primary purpose of this task was to keep participants engaged and motivated to read the sentence pairs attentively, and to be able to check if they in fact did so. Since the analysis of response times was not of interest for the purpose of this experiment, correct and incorrect statements were evenly distributed over experimental items, controls and fillers.

#### Pre-test

Stereotype ratings for 123 potentially stereotypical activities were obtained through an online pre-test administered via Qualtrics. Forty participants (20 male) between the ages of 19 and 32 (*M* = 23.5, *SD* = 2.6) completed the online pre-test, none of whom participated in the eye-tracking experiment. They were asked to indicate the probability of each activity being carried out by a man or a woman on a 7-point Likert scale. Scale direction was varied so that the leftmost point corresponded to female for one half of the participants and to male for the other half. In accordance with previous research [24,40,41], the mean stereotypicality of each activity (with 1 equaling female and 7 equaling male) was calculated, ranging from 1.2 for *beha rechtdoen* ‘adjusting bra’ to 6.5 for *snor scheren* ‘shaving moustache’. From these 123 activities, 16 activities with SD < 1.0 were chosen for each of the three stereotype categories in the eye-tracking experiment. An additional twelve neutral activities with SD < 1.0 were chosen for the control items. All chosen female activities had scored a rating of 3 or lower, male activities had scored a rating of 5 or higher, and neutral stereotypes were centered around 4. The chosen female and male stereotypes were similar in strength; the mean for female stereotypes (*M* = 2.23, *SD* = 0.5) and for male stereotypes (reversed *M* = 2.45, *SD* = 0.38) did not significantly differ (*t* = −1.35, *p* = 0.186) (see S1 Table for all used activities and their pre-test ratings).

All chosen activities were deemed likely to evoke a distributive rather than a collective reading, the former being associated with exhaustive pairing. This means that *each* individual is paired with *one* unique item, there being as many individuals as items. The collective reading, on the other hand, results in an interpretation in which *all* individuals are related to *one* unique item. Consider the following example for illustration:

(6) Everyone was brushing his teeth.

While being ambiguous, the sentence will probably be interpreted in the way that each individual who is brushing teeth is brushing *their own* teeth, this distributive reading being necessary in order for the use of the masculine generic to be felicitous. The other available, but unlikely reading is that everyone is brushing the teeth of one male individual (i.e., the collective reading), in which case the masculine generic reading of the pronoun is not available. Three native speakers of Dutch checked the selection of activities for the eye-tracking experiment and deemed it unlikely for the collective reading to be evoked by any of the items.

### Apparatus and procedure

The experiment was conducted at the Utrecht Institute of Linguistics Lab at Utrecht University, using the EyeLink 1000 remote desktop eye-tracker and the experiment display software ZEP [42]. The participants’ right eye was sampled at 500Hz, but viewing was binocular. The stimuli were presented in a sound-attenuated booth on a 1024×768 monitor, approximately 60cm away from the participant. The stimuli were presented using a medium monospaced font. All participants were tested individually. Upon arrival, participants were informed about the procedure and asked to read the instructions, which were presented on screen. The eye-tracker was fine-tuned to the participant’s eyes, and a calibration and a similar validation procedure followed, during which participants had to fixate a random sequence of dots through 12 positions on the screen. After a practice trial featuring three sentence pairs with two of them being followed by a statement that required a response, participants were given the option to ask clarification questions. After another calibration and validation procedure, the main part of the experiment followed. Stimuli were presented pseudo-randomly, with a maximum of three experimental items following each other and a maximum of two experimental items from the same condition following each other. A drift-check was displayed before all 96 sentence pairs. After the eye-tracking experiment, participants answered the exit question, probing them for the purpose of the experiment. Participants were then paid for their participation.

### Data analysis

First, the fixation pattern of each item was manually checked for each participant. When a systematic and unambiguous shift of all fixations had occurred, these fixations were reassigned to the corresponding regions in accordance with lab recommendations. Furthermore, if the first fixation did not fall on the first word, but the second fixation did, the first fixation was deleted to be able to calculate reading time measures appropriately. After this initial clean-up phase, four reading time measures were calculated: *first fixation duration*, *first gaze duration*, *regression path duration* and *total fixation duration*. *First fixation duration* is the duration of the first fixation in a particular region. *First gaze duration* comprises all fixations in a region before it is left in a forward or backward direction. *Regression path duration* is the sum of fixations in a particular region including regressions to earlier parts of the text until the region is left in a forward direction. *Total fixation duration* comprises all fixations in a particular region, thus including regressions back to that region. An increase in any of these reading time measures is assumed to reflect an increase of the cognitive processing load in a particular region [43,44]. The example in (7) illustrates how experimental items were divided into separate regions for the analysis.

(7) [Iedereen]_1_ [was zijn]_2_ [tanden aan het poetsen]_3_. [Zo was]_4_ [ook Daphne]_5_ [zich aan het klaarmaken]_6_ [om naar bed te gaan.]_7_
  *[Everyone]*_*1*_ *[was his]*_*2*_ *[teeth brushing]*_*3*_. *[So was]*_*4*_ *[also Daphne]*_*5*_ *[getting ready]*_*6*_ *[to go to bed*.*]*_*7*_
  *Everyone was brushing his teeth*. *Daphne*, *too*, *was getting ready to go to bed*.

Region 5 was the primary region of interest and consisted of the proper name preceded by *ook* ‘too’. The decision to include *ook* in this region was made to reduce the probability of the primary region being skipped. This decision is licensed by previous research showing that semantic information is processed parafoveally six to eight characters to the right of the fixated word [45,46]. Region 6 functioned as a spillover region and varied in length between three and four words, depending on the item. If the total character length of the first three words after the proper name counted less than 13 characters, a fourth word was added in order to reduce variability in the region’s length.

For the controls, the division into regions was done the following way, with the primary region of interest being the proper name including the preceding *ook* and the spillover region being defined in the same way as for the experimental items:

(8) [Iedereen was een treinkaartje aan het kopen.]_1_ [Zo was]_2_ [ook Amber]_3_ [bij het loket in]_4_ [de rij gaan staan.]_5_
  *[Everyone was buying a train ticket*.*]*_*1*_ *[So was]*_*2*_ *[also Amber]*_*3*_ *[at the counter]*_*4*_ *[standing in line*.*]*_*5*_
  *Everyone was buying a train ticket*. *Amber*, *too*, *was standing in line at the counter*.

For both experimental and control items, skipped regions were treated as missing data, and log transformations were performed to correct for a positive skew in the control and the experimental data. Observations that were at least 2.5 standard deviations above or below both the condition’s and the region’s mean were excluded.

We modeled the four different reading time measures on the region of interest and the spillover region using linear mixed-effect models. This was done by means of the *lmer* function from the *lme4* package in R [47]. Model selection was done as follows. Committing to a hypothesis-driven approach, *stereotype*, *continuation* and the interaction between the two were included in every model. For control items, *continuation* served as the only initial fixed effect. These categorical variables were coded using sum contrasts. *Female continuation* was coded as 1, *male continuation* was coded as −1. As *stereotype* is a three-level factor, two different contrasts were defined, one contrasting the *female* and the *neutral* level (*female* = 1, *male* = 0, *neutral* = −1), the other contrasting the *male* and the *neutral* level (*female* = 0, *male* = 1, *neutral* = −1). The full random structure permitted by the design [48,49] was initially included as well. Following Bates et al. [50], the random structure was then simplified if there were signs of overparameterization (i.e., when the maximal model failed to converge and/or PCA revealed overparameterization). Simplification was done first by suppressing the correlation parameters. When the PCA still pointed towards overparameterization, the smallest and thus least important variance component was dropped from the model and the PCA was repeated. In a final step, insignificant variance components were dropped making use of Likelihood ratio tests as described by Bates et al. [50]. If removing a variance component significantly decreased the model fit, it was included in the final model. This procedure resulted in all models containing random intercepts for items and subjects only. After the appropriate random effects structure was identified, it was tested using Likelihood Ratio tests whether adding *participant gender* as a fixed effect (both as a single effect only or allowing *participant gender* to interact with *stereotype* and *continuation*) significantly improved the model, in which case *participant gender* was added to the final model. *Participant gender* was coded as 1 for *female* and −1 for *male*. Previous studies on role nouns did not find evidence that men and women differ in their processing of masculine generics [12,25,26], but we decided to control for the possibility nonetheless as effects of participant gender have previously been reported by some studies into pronouns using more explicit methods [14,21]. As models lacking random slopes are often criticized for being anticonservative [48], the conclusions drawn from the final model were compared against those permitted by the model with the most complex random structure that converged. Any discrepancies between significant betas are reported. Following Wald’s criterion, an effect within a model was deemed significant when the absolute t-value exceeded 1.96 [51,52]. P-values were obtained using the normal approximation to the t-statistic. Note, however, that calculating p-values for mixed-effect models is nontrivial and subject to debate [48,53]. Thus, while p-values are reported below, the interpretation of the effects is first and foremost based on the obtained t-values. Significant t-values are reported for the best models.

## Results

Eight out of 92 participants correctly guessed the purpose of the experiment, and their data were therefore excluded from further analysis. Seven of these participants correctly indicated that the experiment investigated a mismatch between the gender stereotype and the gender of an individual (criterion (a)). One participant correctly guessed that the experiment investigated a mismatch between the gender suggested by the masculine generic and the gender of an individual (criterion (b)). Furthermore, the data from one participant were excluded from analysis due to poor quality, as fixations were shifted in an unsystematic manner and could not unambiguously be assigned. In addition, the data of one participant were excluded, because they indicated after the experiment that they were dyslexic. Data of the remaining 82 participants (38 males, age range 18–51, *M* = 22.89, *SD* = 4.87) were analyzed.

### Experimental items

The removed outliers constituted the following percentages of the total data points: 2.6% for first fixation duration, 1.5% for first gaze duration, 1.1% for regression path duration and 1.1% for total fixation duration.

The mean reading times for the proper names and spillover region can be seen in Table 2.

**Table 2:** Mean reading times per condition for the proper name region and spillover region.

		Reading time measure							
		FFdur		FGdur		RPdur		TFdur	
		*M*	*SD*	*M*	*SD*	*M*	*SD*	*M*	*SD*
**Proper name**									
*stereotype*	*continuation*								
neutral	female	180	50	222	107	397	264	405	255
neutral	male	181	51	223	106	389	252	393	250
male	female	187	53	235	116	385	241	427	270
male	male	181	50	243	129	377	231	422	268
female	female	185	55	225	107	362	222	419	291
female	male	185	57	241	130	405	256	456	289
**Spillover**									
*stereotype*	*continuation*								
neutral	female	196	57	496	266	580	338	671	378
neutral	male	195	56	475	265	572	350	665	428
male	female	203	67	457	261	601	427	688	424
male	male	207	66	479	284	572	367	688	430
female	female	194	53	466	263	559	346	655	394
female	male	201	59	463	247	559	343	663	397

Means (M) and standard deviations (SD) given in milliseconds for first fixation duration (FFdur), first gaze duration (FGdur), regression path duration (RPdur) and total fixation duration (TFdur).

#### Primary region of interest: The proper name

The earliest significant effects were found for first gaze duration on the proper name. Adding *participant gender* to the model significantly improved the model fit, and the effect of gender itself was significant (*β* = −0.05, *SE* = 0.02, *t* = −2.24, *p* = 0.025, 95% bootstrapped CI of *β* [−0.09; −0.007]), suggesting that on average the first gaze duration of female participants was significantly shorter. Furthermore, there was a significant effect of *stereotype*. First gaze duration was significantly longer after stereotypically male (*M* = 239.2, *SD* = 122.4) than after stereotypically neutral contexts (*M* = 222.4, *SD* = 106.3) (*β* = 0.03, SE = 0.01, *t* = 2.56, *p* = 0.010, 95% bootstrapped CI of *β* [0.005; 0.048]). The comparison between female (*M* = 233.2, *SD* = 119.8) and neutral stereotype contexts, on the other hand, was not significant (*β* = 0.005, SE = 0.01, *t* = 0.32, *p* = 0.750, 95% bootstrapped CI of *β* [−0.016; 0.022]).

**Fig 1:**
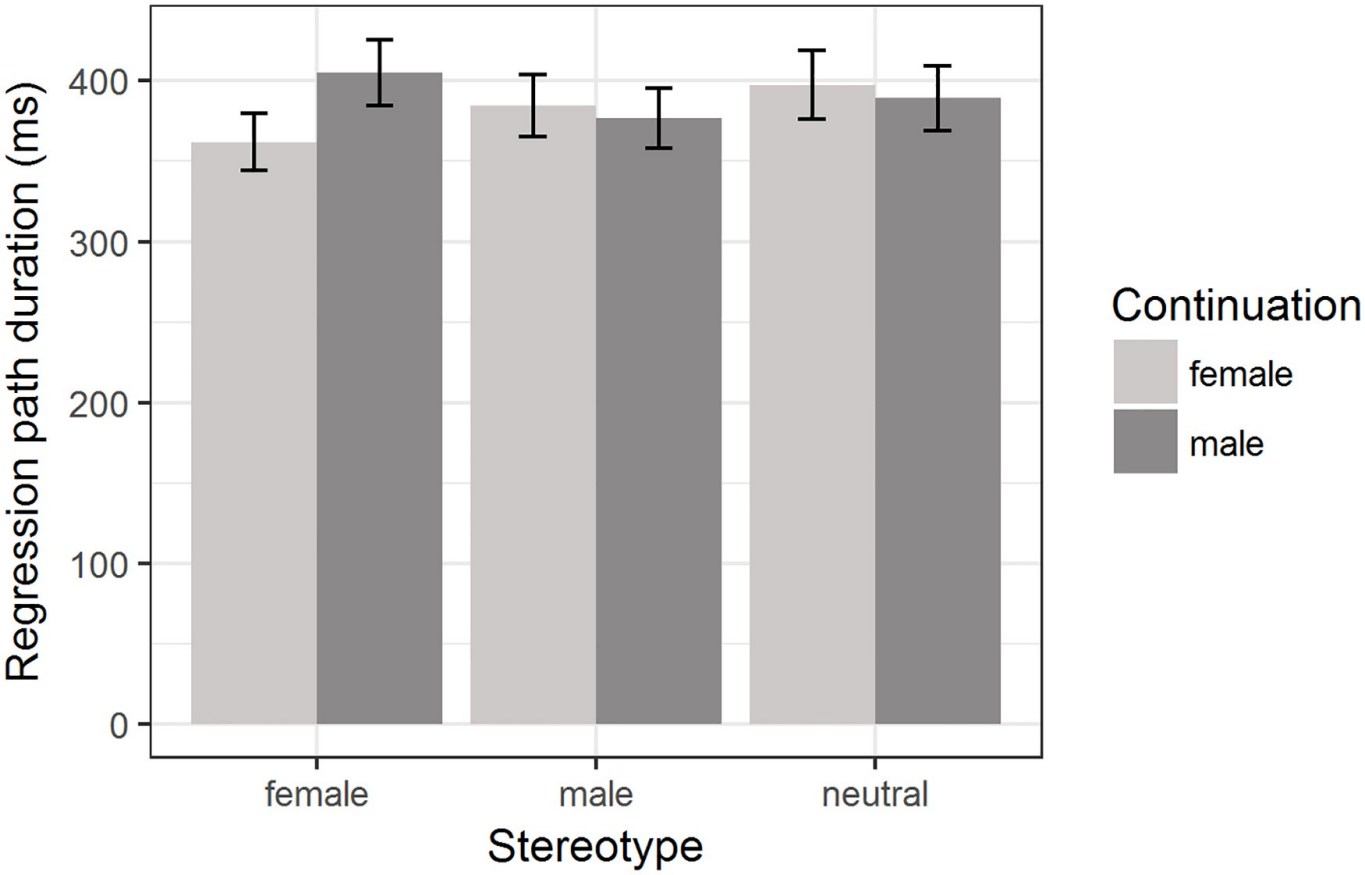
Regression path duration for male and female proper names in male, female and neutral stereotype contexts. Condition means are given in milliseconds. Error bars represent 95% confidence intervals.

The model for regression path duration revealed a significant interaction effect between *stereotype* and *continuation* when contrasting the female and neutral stereotype contexts (*β* = −0.04, SE = 0.01, *t* = −3.25, *p* = 0.001, 95% bootstrapped CI of *β* [−0.06; −0.014]). As can be seen in Fig 1, female proper names were read significantly faster compared to male proper names in female stereotype contexts, but no such difference was found in neutral stereotype contexts. The interaction effect between *stereotype* and *continuation* when contrasting the male and neutral stereotype contexts, on the other hand, was not significant (*β* = 0.02, SE = 0.01, *t* = 1.92, *p* = 0.055, 95% bootstrapped CI of *β* [−0.001; 0.044]). To summarize, encountering a male proper name (i.e., *continuation* = male) in a female stereotype context led to a significant increase in regression path duration, but encountering a female proper name after a male stereotype context did not. Furthermore, no difference between proper names was found for neutral stereotype contexts. S1 Fig shows the condition means for the log-transformed data and can be found in the supplementary material.

Thus, participants spent more time on the proper name itself and reread previous portions of the text when they read sentence pairs as in (9):

(9) *Iedereen was zijn yogaoefeningen aan het doen*. *Zo was ook Peter goed bezig met een oefening*.
  ‘Everyone was doing his yoga exercises. Peter, too, was engaged in an exercise.’

However, sentence pairs such as in (10), featuring a woman engaging in a stereotypically male activity, did not lead to a significant increase in regression path duration:

(10) *Iedereen was zijn voetbaltrucs aan het oefenen*. *Zo was ook Laura al urenlang met de bal bezig*.
  ‘Everyone was practicing his soccer tricks. Laura, too, had been playing with the ball for hours.’

No evidence for a male bias induced by *zijn* was found: There was no significant increase of regression path duration for female proper names compared to male proper names in the neutral stereotype context, where such a male bias should be easily detectable, nor in the female and male stereotype context.

**Fig 2:**
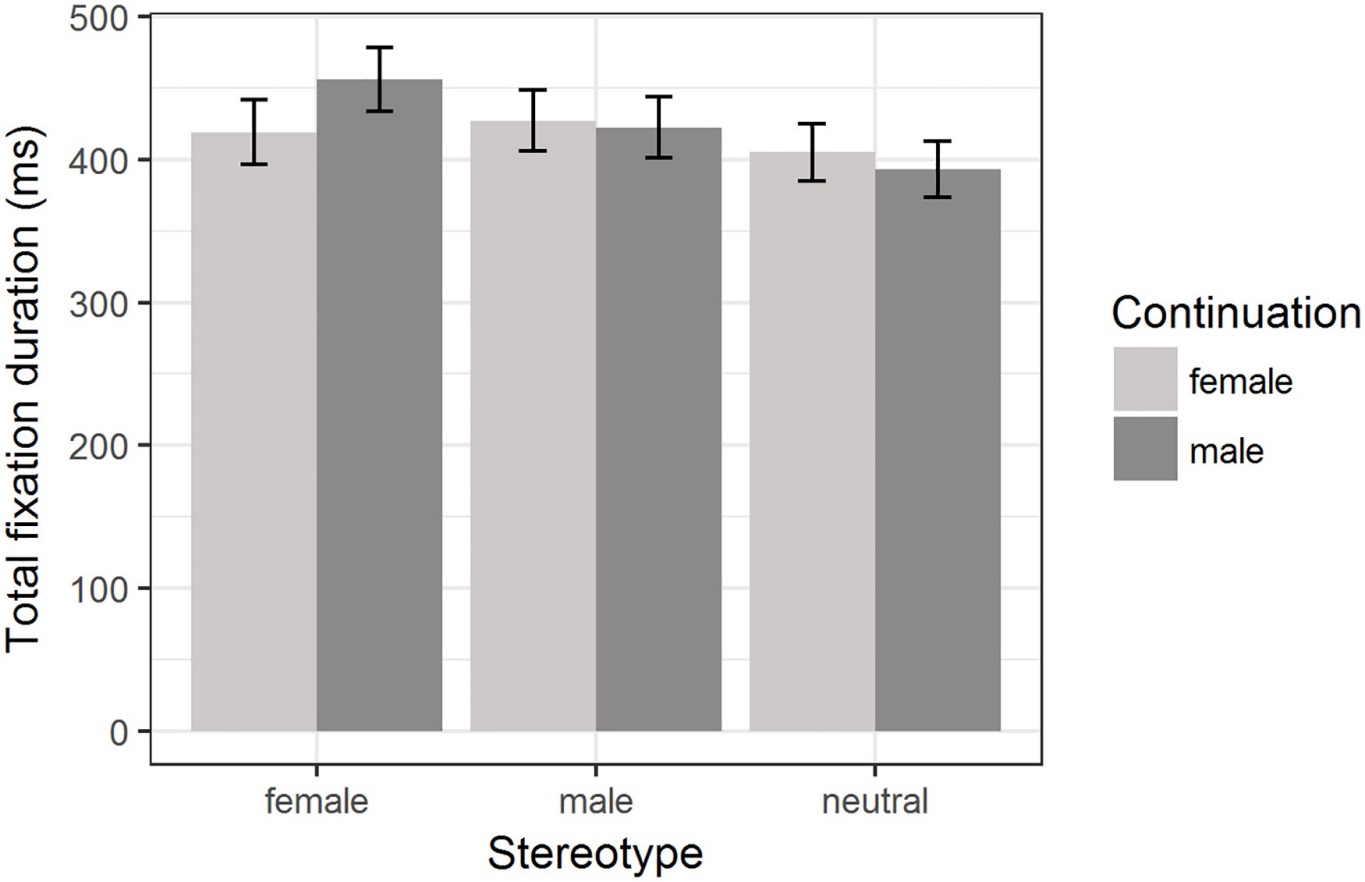
Total fixation duration for male and female proper names in male, female and neutral stereotype contexts. Condition means are given in milliseconds. Error bars represent 95% confidence intervals.

A similar pattern arose for total fixation duration. There was a significant effect of *participant gender*, with female participants’ total fixation duration being significantly shorter overall (*β* = −0.11, *SE* = 0.03, *t* = −3.8, *p <* 0.001, 95% bootstrapped CI of *β* [−0.166; −0.049]). Furthermore, similar to the results for the regression path duration, the interaction effect between *stereotype* and *continuation* was significant when contrasting the female and neutral stereotype contexts (*β* = −0.04, SE = 0.01, *t* = −3.61, *p* < 0.001, 95% bootstrapped CI of *β* [−0.069; −0.02]), but not when contrasting the male and neutral stereotype contexts (*β* = 0.02, SE = 0.01, *t* = 1.89, *p* = 0.058, 95% bootstrapped CI of *β* [−0.001; 0.045]), as can be seen in Fig 2. See S2 Fig in the supplementary material for a figure showing the log-transformed data.

Thus, as with the results for regression path duration, encountering a male proper name in a female stereotype context led to a significant increase in reading times compared to female proper names, while no such difference between proper names was found for neutral and male stereotype contexts. No evidence for the presence of a male bias induced by the masculine generic *zijn* was found, as there was no significant increase in total fixation duration for female continuations in any of the contexts.

#### Spillover region

Two significant main effects emerged for first fixation duration in the spillover region. There was a significant main effect of *continuation*, suggesting that first fixations for female continuations (*M* = 197.5, *SD* = 59.4) and male continuations (*M* = 201.1, *SD* = 59.4) differed significantly (*β* = −0.01, SE = 0.004, *t* = −2.24, *p* = 0.025, 95% bootstrapped CI of *β* [−0.017; −0.001]). Note, however, that this rather small difference of 3.6 milliseconds is hardly meaningful and mainly driven by the small standard error. We further found a significant main effect of *stereotype* when comparing the male (*M* = 205, *SD* = 66.6) and neutral (*M* = 195.4, *SD* = 56.3) stereotype levels, with first fixations being significantly longer after male stereotype contexts (*β* = 0.02, SE = 0.01, *t* = 2.12, *p* = 0.034, 95% bootstrapped CI of *β* [0.001; 0.041]). There were no significant effects for first gaze duration.

For regression path duration, the only significant effect was *participant gender*. Male participants showed a higher regression path duration in the spillover region than female participants (*β* = −0.07, SE = 0.03, *t* = −2.27, *p* = 0.023, 95% bootstrapped CI of *β* [−0.122; −0.009]).

**Fig 3:**
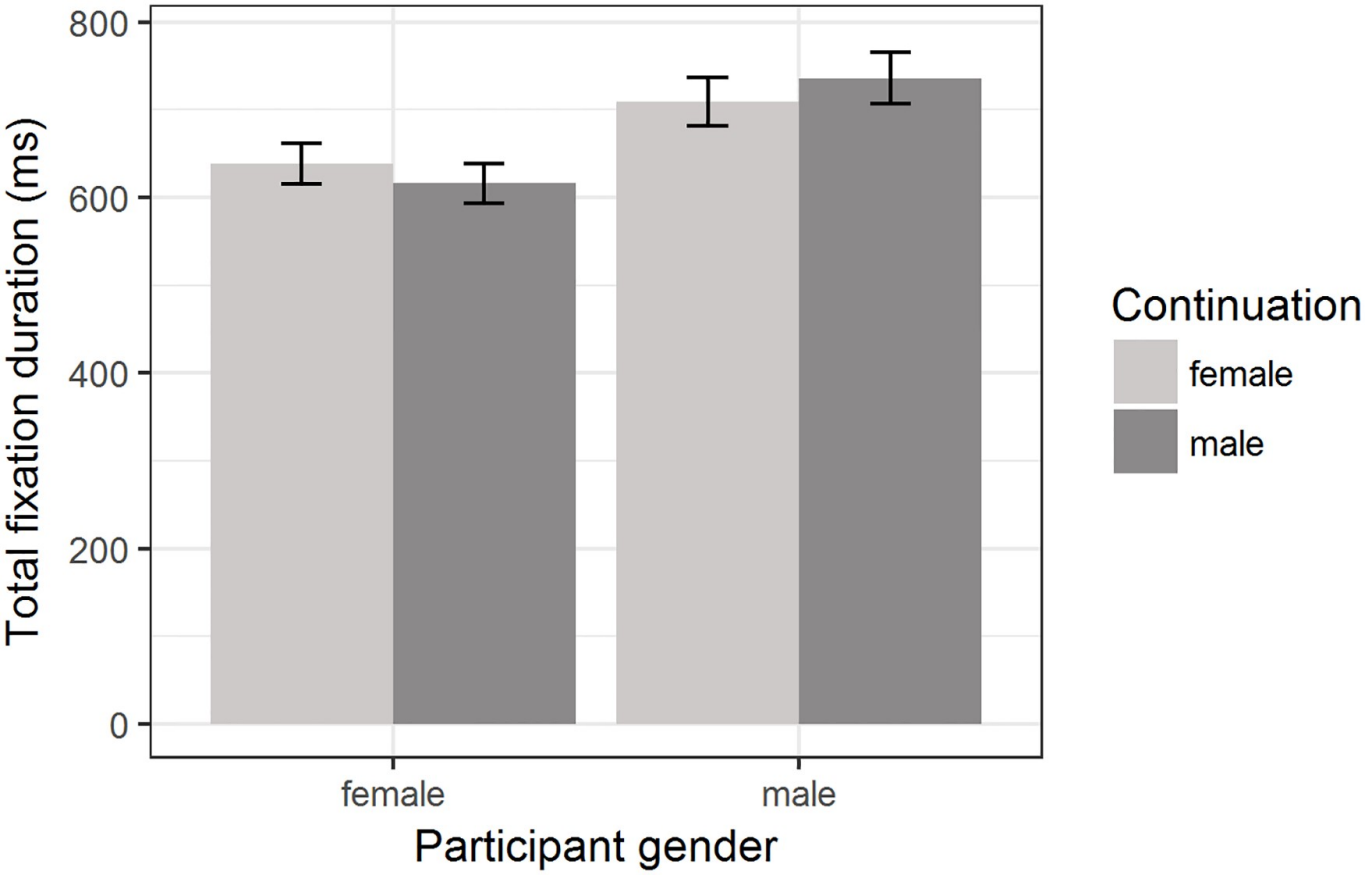
Total fixation duration in the spillover region for male and female proper names, shown separately for male and female participants. Condition means are given in milliseconds. Error bars represent 95% confidence intervals.

For total fixation duration in the spillover region, there was again a significant main effect of participant gender (*β* = −0.07, SE = 0.03, *t* = −2.52, *p* = 0.012, 95% bootstrapped CI of *β* [−0.129; −0.016]), but more interestingly there was a significant interaction effect between *participant gender* and *continuation* (*β* = 0.02, *SE* = 0.01, *t* = 2.13, *p* = 0.033, 95% bootstrapped CI of β [0.002; 0.032]). Female participants showed slightly higher reading times in the spillover region after a female proper name (*M* = 638.9, *SD* = 379) than after a male proper name (*M* = 615.9, *SD* = 376) in the case of total fixation duration. The pattern was reversed for male participants, with a slightly higher total fixation duration after male proper names (*M* = 736, *SD* = 454.3) than after female proper names (*M* = 709.1, *SD* = 417.9), as can be seen in Fig 3. Descriptively, this can be most clearly seen for neutral stereotype contexts (see Table 3), but the three-way interaction supporting this was not borne out statistically (*β* = −0.02, *SE* = 0.01, *t* = −1.8, *p* = 0.07, 95% bootstrapped CI of β [−0.04; 0.002] when contrasting neutral and male stereotype contexts). Note that while the main interaction effect between *participant gender* and *continuation* is significant, the simple effect of continuation within female and male participants, respectively, is not significant as reflected by the widely overlapping confidence intervals in both participant groups. See S3 Fig for the transformed data.

**Table 3:** Mean total fixation duration per condition for the spillover region, shown separately for male and female participants.

		Total fixation duration			
		Male participants		Female participants	
		*M*	*SD*	*M*	*SD*
Spillover					
*stereotype*	*continuation*				
neutral	female	675	401	667	356
neutral	male	756	483	585	354
male	female	738	449	645	396
male	male	732	453	649	405
female	female	714	400	605	382
female	male	719	424	613	365

Means (M) and standard deviations (SD) for total fixation duration given in milliseconds.

### Control items

As with the experimental stimuli, all observations 2.5 standard deviations above or below condition and region mean were removed. Based on this criterion, 2.8% of the observations were removed for first fixation duration, 2% for first gaze duration, 1.2% for regression path duration and 0.8% for total fixation duration.

The mean reading times for the proper names and spillover region of the controls can be found in S2 Table.

For all the reported models, the best random structure was identified as featuring random intercepts for both participants and items, but no random slopes for any of the fixed effects. No significant effect of *continuation* was observed in any of the models. Similar to the experimental items, a significant effect of *participant gender* was occasionally observed, with male participants taking longer than female participants. On the proper name, this effect of gender was observed for first gaze duration (*β* = −0.05, *SE* = 0.02, *t* = −2.18, *p* = 0.030, 95% bootstrapped CI of *β* [−0.098; −0.009]), for regression path duration (*β* = −0.07, *SE* = 0.04, *t* = 2, *p* = 0.046, 95% bootstrapped CI of *β* [−0.145; −0.004) and total fixation duration (*β* = −0.11, *SE* = 0.03, *t* = −3.75, *p <* 0.001, 95% bootstrapped CI of *β* [−0.179; −0.055]). In the spillover region, this effect was observed for first gaze duration (*β* = −0.11, *SE* = 0.03, *t* = −2.12, *p* = 0.034, 95% bootstrapped CI of *β* [−0.102; −0.004]) and for total fixation duration (*β* = −0.09, *SE* = 0.03, *t* = −2.86, *p* = 0.004, 95% bootstrapped CI of *β* [−0.144; −0.032]).

## Discussion

We conducted an eye-tracking experiment to test whether the Dutch masculine generic *zijn* ‘his’ leads to a male bias despite being generically-intended. By presenting *zijn* in female, male and neutral stereotype contexts, we could further test whether this hypothesized male bias persisted across contexts or—alternatively—was overruled by it.

### No evidence of a male bias

Against our expectations, we found no evidence that the grammatical gender of *zijn* biased participants towards a male interpretation. Thus, in the absence of other gender cues, participants’ reading times of male and female proper names did not significantly differ despite the use of a grammatically masculine pronoun. Mostly older offline research on English masculine generic pronouns had previously found evidence for a male bias caused by masculine generic pronouns [14,18,19,21,22,54], but the present study is the first to thoroughly investigate the processing of masculine generic pronouns. This difference in methodology and consequently a difference in the measured construct could explain this. As outlined earlier, offline methods might not reflect the presence of an automatic gender inference induced by the grammatical gender of the pronoun. Any observed male bias might instead be caused by participants’ conscious reasoning. In our eye-tracking experiment, however, we did not give participants the opportunity to ponder on how the masculine generic *zijn* ‘his’ is intended—generic or male-specific; reading times reflected the *immediate* processing of the masculine generic. It is therefore possible that the male bias of the generically-intended possessive pronouns is amplified—or only comes to light—when allowing for more strategic and explicit responses.

Another reason for not finding a male bias might lie in the lower salience of the possessive pronoun compared to role nouns. In addition to being only a subpart of a larger noun phrase, this noun phrase also appeared as a direct object. The masculine generic was further used anaphorically, thus as referring back to previously introduced referents. Conversely, the online experiments testing the male bias of masculine generic role nouns usually make use of stimuli in which the role noun is introduced as the subject of the sentence and as a noun phrase in its own right [12,25,26,28]. Thus, the grammatical gender of a possessive pronoun might be more easily overlooked due to its lower salience and anaphoric use.

In a similar vein, it is possible that in Dutch, particularly, the grammatical gender of the generically-used possessive pronoun affects processing only to a limited degree. The Dutch grammatical gender system has recently undergone a process of resemanticization [55]. Dutch as spoken in the Netherlands only distinguishes between common and neuter grammatical gender on nouns, but retained the original three-way distinction between masculine, feminine and neuter on pronouns. Thus, there is a mismatch between the nominal and pronominal gender system. Audring’s analysis [55] shows that this mismatch is resolved by using the masculine grammatical gender as a sort of default when something or someone is highly salient as an individual. The feminine gender, however, is only used when referring to female individuals or to a few feminine animals [55]. Thus, the generic function of the masculine grammatical gender is omnipresent in Dutch and might be more readily available compared to other languages.

Another Dutch peculiarity might have added to the generic potential of the possessive pronoun specifically. At the surface code level, the possessive pronoun *zijn* shows structural overlap with two other pronouns: *zij* ‘they’ and *zij* ‘she’. Past research on visual word recognition has shown that partial words prime their targets [56,57]. Thus, it is possible that the activation of female *zij* ‘she’ and underspecified *zij* ‘they’ counteracted a male bias induced by masculine *zijn* ‘his’. Previous research on German role nouns as masculine generics has shown that subtle morphological relations may attenuate the male bias of masculine generics. More specifically, Garnham et al. [28] found that the presence of the German pronoun *sie* ‘they’ can attenuate the male bias of role nouns as masculine generics due to its resemblance with feminine *sie* ‘she’. However, we deem it unlikely that this structural overlap overwrote expectations based on grammar, as *zij* ‘she’ and *zij* ‘they’ would both be ungrammatical when used instead of the possessive pronoun *zijn* ‘his’ in our stimuli.

Finally, as we outlined before, we believe that masculine generic pronouns might work inherently differently from masculine generic role nouns, for which a male bias has been found repeatedly [12,25,26,28,29]. The generic potential of pronouns might be higher overall, as the very same masculine generic token is used generically over and over again. For role nouns, it is only the pattern type that is repeated and differences in the frequency of the feminine compared to the masculine form of an individual role noun might determine to what extent a specific masculine role noun can be interpreted as generic [36]. Future research is necessary to replicate the present results both in Dutch and in other languages to determine whether masculine pronouns can truly be interpreted generically, whether this is only true for Dutch or even only for the possessive pronoun in Dutch. We have clearly shown that there is a necessity to expand research into masculine generics to other types than role nouns in order to understand their effect on language processing and beyond.

### Gender stereotypes and attitudes towards their violation affect language processing

While the pronoun did not significantly affect sentence processing, gender stereotype violations did. When a male proper name was mentioned after a female context, a significant increase in regression path duration and total fixation duration occurred; when a female proper name was mentioned after a male context, no such increase occurred. Thus, a man engaging in a female activity led to processing difficulties while the reverse was not the case. Theoretically, this could be due to a difference in the strength of the female and male stereotypes used in our experiment. However, these had been carefully pre-tested drawing from the same population, and they were carefully selected based on their mean and standard deviation (see the Materials and method section for details). Further backing up our claim that the asymmetry we found is meaningful is the fact that this asymmetry has been found in two other eye-tracking studies [58,59] and two priming experiments investigating response times [60] and event-related potentials [61].

Cacciari and Padovani [60] adapted their experiment from the priming experiment by Banaji and Hardin [62], and provided linguistic evidence for an asymmetrical response to gender stereotype violations. The authors tested Italian role nouns which, when presented in bare form, could refer to either men or women grammatically (e.g., *emigrante* ‘emigrant’), but they differed in terms of gender stereotype between male and female. An additional neutral baseline condition was used. The role nouns functioned as primes for the pronouns *lui* ‘he’ and *lei* ‘she’, the grammatical gender of which participants had to indicate as fast as possible. Cacciari and Padovani [60] found that participants generally responded faster when the pronoun gender and the gender stereotype matched. They also found that a mismatch led to an increase in response times compared to the neutral baseline, but *only* for stereotypically female role nouns being followed by a masculine pronoun. When *lei* ‘she’ was the target, no difference was found for response times after a stereotypically male and neutral prime. Siyanova-Chanturia et al. [61] employed a similar design, additionally measuring event-related potentials. They found an N400-like effect when a masculine pronoun was presented after a stereotypically female prime, but not when a feminine pronoun was presented after a stereotypically male prime. Reali et al. [59], in an attempt to disentangle the effect of a role noun’s grammatical gender and its stereotypicality in German, used descriptions of role nouns in their eye-tracking stimuli instead of the role nouns themselves (e.g., *makes appointments*, *deals with the correspondence in an office* as a description of *secretary*). They found that following female role noun descriptions, participants had difficulty when the described person (e.g., the secretary) was revealed to be a man. However, in the reverse condition, when a woman was revealed to work in a stereotypically male job, reading times did not increase. One of the possible explanations the authors offered for this lies in the grammatical gender of the described role nouns. Reali et al. [59] argue that the role noun descriptions might have activated the role noun itself and therefore its grammatical gender. For stereotypically male role noun descriptions, the grammatically masculine role noun would have been activated. For stereotypically female role nouns, the grammatically feminine role noun would have been activated. Since grammatically masculine role nouns can at least in theory be interpreted as generic in German, participants might not have experienced processing difficulties when reading about a female individual in a male stereotype context. However, grammatically feminine role nouns can only be used to refer to female individuals. Thus, when a male individual is mentioned, processing difficulties occur in a female stereotype context. Reali et al. [59] interpreted the fact that this asymmetry had up to that point only been found in languages distinguishing between masculine and feminine grammatical gender as support for this explanation. However, this idea is not compatible with our own results as we did not use descriptions of role nouns, which could have activated the role nouns’ grammatical gender, but instead we used a myriad of activities, most of which cannot be captured by a specific role noun. Furthermore, in a follow-up experiment applying the same method to English role nouns, Reali et al. [58] found the same asymmetry. As English does not mark grammatical gender on nouns (with a few potential exceptions such as *waitress* carrying the suffix–*ess* for female agents), the explanation of the grammatical gender of described role nouns being automatically activated can definitely be ruled out.

Instead, we propose that discourse expectations about upcoming referents are not only guided by the typicality of role nouns or activities, but also by the acceptability of violating these stereotypes. This is in line with research in social psychology and sociology showing that men violating gender roles are disapproved of more than women violating gender roles [63–66]. The first of the two main competing explanations roots this asymmetry in a difference between men and women’s social status. For example, Feinman [64] argued that the male role enjoys higher status than the female role. Therefore, men engaging in stereotypically female behavior are seen as seeking downward mobility and decreasing in status, whereas women engaging in stereotypically male behavior seek upward mobility, which constitutes an increase in status. As Feinman [64] put it: “it is worse to be a sissy than a tomboy”. Alternatively, the sexual orientation hypothesis suggests that men who exhibit feminine behavior are more likely to be thought of as homosexual than women behaving in a masculine manner, this, too, being a group with lower social status [63]. Whatever the exact cause of the phenomenon that men’s gender roles are more rigid, it is reflected in online processing. This adds to previous research showing that social stereotypes are rapidly used in language processing [67,68].

We did not, however, find evidence of any discrepancy in social status between men and women being reflected in language processing. While it was not the focus of our study to test whether a more general male bias in language processing exists even in the absence of masculine generics (i.e., the people = male bias), we had to control for the possibility that men are in fact seen as the prototypical humans, as has been suggested by some studies [17,25,26]. This could have possibly led to the generally faster processing of contexts featuring male individuals, but we did not find evidence for this.

### Participant gender and other puzzles

An interaction effect including participant gender emerged in the spillover region: whether a male or female proper name was presented had a differential effect on male and female participants. Female participants’ total fixation duration slightly increased when a female proper name had been mentioned. For men, the pattern was reversed. Thus, participants seemed to pay slightly more attention to the spillover region when the protagonist shared their gender. Due to careful counterbalancing, this interaction effect cannot be due to differences in the stimuli. Surprisingly, this effect was not found for the maximally similar control items. Future research will have to determine whether participants’ interest in stimuli featuring protagonists of their own gender was a statistical fluke or rather constitutes a robust pattern.

Participant gender affected our results in yet another way. On many reading measures both on the proper names and spillover regions for experimental items and controls, we found that male participants showed increased reading times overall. A similar effect was found in an eye-tracking experiment by Reali et al. [58]. Furthermore, Osterhout et al. [33] and Siyanova-Chanturia et al. [61] found that women’s electrophysiological responses to gender stereotype violations can be stronger as reflected in larger ERP deflections. However, the effect in our experiment seems to be of a more general nature as it was found not only in response to gender stereotype violations, but also in their absence. This suggests that the effect we found is likely due to a more general difference: Women are often found to be better and faster readers than men [69,70].

We further found that first gaze duration was increased on the proper name—regardless of the gender of the referent—after male stereotype contexts compared to neutral stereotype contexts. The same was found for first fixation duration in the spillover region. We interpret these as spillover effects from the stereotype context itself. This could be due to an elaborative gender inference being made based on the stereotype context [30–32]. However, no significant increase in reading time was found for female stereotype contexts compared to neutral contexts, which renders this explanation unlikely. We therefore believe this increase to be due to item-inherent frequency effects: The combined nouns and verbs used for neutral stereotype contexts are presumably more frequent than the male stereotypes.

## Conclusion

To conclude, we found no evidence for a male bias induced by the generically-intended masculine pronoun *zijn* ‘his’. This emphasizes the importance of considering different types of masculine generics cross-linguistically in order to understand how they affect language processing. We showed that gender inferencing in language goes beyond the mostly occupational and social stereotypes carried by role nouns, but pertains to stereotypical activities, too. Furthermore, our results indicate that discourse expectations are not only guided by the strength of gender stereotypes themselves, but also by the severity of flouting them.

## Supporting information

S1 Appendix List of experimental stimuli and control items. (PDF)

S1 Table Ratings of activities used in stimuli and controls. Ratings range from 1 to 7, with 1 corresponding to female and 7 to male.
(PNG)

S2 Table Mean reading times for the control items per condition, for the proper name region and spillover region. Means (M) and standard deviations (SD) given in milliseconds for first fixation duration (FFdur), first gaze duration (FGdur), regression path duration (RPdur) and total fixation duration (TFdur).
(PNG)

S1 Fig Regression path duration for male and female proper names in male, female and neutral stereotype contexts. Condition means are given in log milliseconds. Error bars represent 95% confidence intervals.
(TIF)

S2 Fig Total fixation duration for male and female proper names in male, female and neutral stereotype contexts. Condition means are given in log milliseconds. Error bars represent 95% confidence intervals.
(TIF)

S3 Fig Total fixation duration in the spillover region for male and female proper names, shown separately for male and female participants. Condition means are given in log milliseconds. Error bars represent 95% confidence intervals.
(TIF)

## References

[1] Pauwels A. Women changing language. London & New York: Longman; 1998 10.1017/S0047404500242048

[2] Hellinger M, Bußmann H, editors. Gender across languages The linguistic representation of women and men. Amsterdam: 2001 10.1075/impact.9

[3] Dale Van. Groot woordenboek van de Nederlandse taal. 15th ed Utrecht & Antwerpen: Van Dale publishers; 2015 10.1080/19306962.1951.11786557

[4] Vaessen R. Elke postbezorger zal zich moeten afvragen wat hij kan doen om als geheel sterker te staan. De Volkskrant [newspaper online]. 2017 Sep 28 [cited 2017 Dec 20]. https://www.volkskrant.nl/opinie/elke-postbezorger-zal-zich-moeten-afvragen-wat-hij-kan-doen-om-als-geheel-sterker-te-staan~a4518898/.

[5] Gabriel U, Mellenberger F. Exchanging the generic masculine for gender-balanced forms—The impact of context valence. Swiss J Psychol 2004;63:273–8. 10.1024/1421-0185.63.4.273

[6] Garnham A, Yakovlev Y. The interaction of morphological and stereotypical gender information in Russian. Front Psychol 2015;6:1720:1–12. 10.3389/fpsyg.2015.01720 26635650 PMC4644804

[7] Irmen L, Schumann E. Processing grammatical gender of role nouns: Further evidence from eye movements. J Cogn Psychol (Hove) 2011;23:998–1014. 10.1080/20445911.2011.596824

[8] Braun F, Sczesny S, Stahlberg D. Cognitive effects of masculine generics in German: An overview of empirical findings. Communications 2005;30:1–21. 10.1515/comm.2005.30.1.1

[9] Hamilton MC. Using masculine generics: Does generic He increase male bias in the user’s imagery? Sex Roles 1988;19:785–99. 10.1007/BF00288993

[10] Stahlberg D, Sczesny S, Braun F. Name your favorite musician: Effects of masculine generics and of their alternatives in German. J Lang Soc Psychol 2001;20:464–9. 10.1177/0261927X01020004004

[11] Braun F, Gottburgsen A, Sczesny S. Können Geophysiker Frauen sein? Generische Personenbezeichnungen im Deutschen. Zeitschrift Für Germanistische Linguistik 1998;26:265–83. 10.1515/zfgl.1998.26.3.265

[12] Gygax P, Gabriel U, Sarrasin O, Oakhill J, Garnham A. Generically intended, but specifically interpreted: When beauticians, musicians, and mechanics are all men. Lang Cogn Process 2008;23:464–85. 10.1080/01690960701702035

[13] Bodine A. Androcentrism in prescriptive grammar: singular ‘they’, sex-indefinite ‘he’, and ‘he or she’. Language in Society 1975;4:129–46. 10.1017/S0047404500004607

[14] Moulton J, Robinson GM, Elias C. Sex bias in language use: “Neutral” pronouns that aren’t. Am Psychol 1978;33:1032–6. 10.1037/0003-066X.33.11.1032

[15] Pusch LF. Das Deutsche als Männersprache. Frankfurt Am Main: Suhrkamp; 1984.

[16] Romein-Verschoor A. Over taal en seks, seksisme en emancipatie. De Gids 1975;138:3–29.

[17] Silveira J. Generic masculine words and thinking. Womens Stud Int Q 1980;3:165–78. 10.1016/S0148-0685(80)92113-2

[18] Gastil J. Generic pronouns and sexist language: The oxymoronic character of masculine generics. Sex Roles 1990;23:629–43. 10.1007/BF00289252

[19] Hyde JS. Children’s understanding of sexist language. Dev Psychol 1984;20:697–706. 10.1037/0012-1649.20.4.697

[20] MacKay DG, Fulkerson DC. On the comprehension and production of pronouns. J Verbal Learning Verbal Behav 1979;18:661–73. 10.1016/S0022-5371(79)90369-4

[21] Switzer JY. The impact of generic word choices: An empirical investigation of age- and sex-related differences. Sex Roles 1990;22:69–82. 10.1007/BF00288155

[22] Miller MM, James LE. Is the generic pronoun he still comprehended as excluding women? Am J Psychol 2009;122:483–96. 20066927

[23] Cole CM, Hill FA, Dayley LJ. Do masculine pronouns used generically lead to thoughts of men? Sex Roles 1983;9:737–50. 10.1007/BF00289802

[24] Gabriel U, Gygax P, Sarrasin O, Garnham A, Oakhill J. Au pairs are rarely male: Norms on the gender perception of role names across English, French, and German. Behav Res Methods 2008;40:206–12. 10.3758/BRM.40.1.206 18411544

[25] Irmen L, Roßberg N. Gender markedness of language: The impact of grammatical and nonlinguistic information on the mental representation of person information. J Lang Soc Psychol 2004;23:272–307. 10.1177/0261927X04266810

[26] Irmen L. What’s in a (role) name? Formal and conceptual aspects of comprehending personal nouns. J Psycholinguist Res 2007;36:431–56. 10.1007/s10936-007-9053-z 17372839

[27] Sato S, Gygax P, Gabriel U. Gauging the impact of gender grammaticization in different languages: Application of a linguistic-visual paradigm. Front Psychol 2016;7:140:1–13. 10.3389/fpsyg.2016.00140 26941663 PMC4762989

[28] Garnham A, Gabriel U, Sarrasin O, Gygax P, Oakhill J. Gender representation in different languages and grammatical marking on pronouns: When beauticians, musicians, and mechanics remain men. Discourse Process 2012;49:481–500. 10.1080/0163853X.2012.688184

[29] Misersky J. The effects of grammatical gender on referent processing in German: An ERP Study. Proceedings of the Master’s Programme Cognitive Neuroscience of the Radboud University 2017;12:74–82.

[30] Carreiras M, Garnham A, Oakhill J, Cain K. The use of stereotype gender information in constructing a mental model: Evidence from English and Spanish. Q J Exp Psychol 1996;49A:639–63. 10.1080/713755647 8828401

[31] Garnham A, Oakhill J, Reynolds D. Are inferences from stereotyped role names to characters’ gender made elaboratively? Mem Cognit 2002;30:439–46. 10.3758/BF03194944 12061764

[32] Oakhill J, Garnham A, Reynolds DJ. Immediate activation of stereotypical gender information. Mem Cognit 2005;33:972–83. 10.3758/BF03193206 16496719

[33] Osterhout L, Bersick M, McLaughlin J. Brain potentials reflect violations of gender stereotypes. Mem Cognit 1997;25:273–85. 10.3758/BF03211283 9184479

[34] Zwaan R, Radvansky G. Situation models in language comprehension and memory. Psychol Bull 1998;123:162–85. 10.1037/0033-2909.123.2.162 9522683

[35] Hogeweg L. Word in process. On the interpretation, acquisition, and production of words. vol. 17 Netherlands Graduate School of Linguistics; 2009 10.1080/10489223.2010.509266

[36] De Backer MD, De Cuypere LD. The interpretation of masculine personal nouns in German and Dutch: A comparative experimental study. Language Sciences 2012;34:253–68. 10.1016/j.langsci.2011.10.001

[37] Vervecken D, Hannover B. Yes I can! Effects of gender fair job descriptions on children’s perceptions of job status, job difficulty, and vocational self-efficacy. Social Psychology 2015;46:76–92. 10.1027/1864-9335/a000229

[38] Vervecken D, Hannover B, Wolter I. Changing (S)expectations: How gender fair job descriptions impact children’s perceptions and interest regarding traditionally male occupations. J Vocat Behav 2013;82:208–20. 10.1016/j.jvb.2013.01.008

[39] Nederlandse Voornamenbank. https://www.meertens.knaw.nl/nvb/.

[40] Kennison SM, Trofe JL. Comprehending pronouns: A role for word-specific gender stereotype information. J Psycholinguist Res 2003;32:355–78. 10.1023/A:1023599719948 12845944

[41] Misersky J, Gygax PM, Canal P, Gabriel U, Garnham A, Braun F, et al Norms on the gender perception of role nouns in Czech, English, French, German, Italian, Norwegian, and Slovak. Behav Res Methods Instrum Comput 2014:1–78. 10.3758/s13428-013-0409-z 24163213

[42] Veenker T. The ZEP experiment control application. Computer software, version 1.2. 2012.

[43] Rayner K. Eye movements in reading and information processing: 20 years of research. Psychol Bull 1998;124:372–422. 10.1037/0033-2909.124.3.372 9849112

[44] Rayner K. Eye movements and attention in reading, scene perception, and visual search. Q J Exp Psychol 2009;62:1457–506. 10.1080/17470210902816461 19449261

[45] McConkie GW, Rayner K. The span of the effective stimulus during a fixation in reading. Perception & Psychophysics 1975;17:578–86. 10.3758/BF03203972

[46] Schroyens W, Vitu F, Brysbaert M, D’Ydewalle G. Eye movement control during reading: Foveal load and parafoveal processing. Q J Exp Psychol 1999;52A:1021–46. 10.1080/713755859 10605397

[47] Bates D, Mächler M, Bolker B, Walker S. Fitting linear mixed-effects models using lme4. J Stat Softw 2015;67:1–48. doi: 10.18637/jss.v067.i01

[48] Barr DJ, Levy R, Scheepers C, Tily HJ. Random effects structure for confirmatory hypothesis testing: Keep it maximal. J Mem Lang 2013;68:255–78. 10.1016/j.jml.2012.11.001 24403724 PMC3881361

[49] Barr DJ. Random effects structure for testing interactions in linear mixed-effects models. Front Psychol 2013;4:328:1–2. 10.3389/fpsyg.2013.00328 23761778 PMC3672519

[50] Bates DM, Kliegl R, Vasishth S, Baayen H. Parsimonious mixed models. ArXiv:150604967 [stat.ME] 2015:1–27.

[51] Hox JJ. Applied Multilevel Analysis. Amsterdam: TT Publicaties; 1995.

[52] Quené H, Van den Bergh H. On multi-level modeling of data from repeated measures designs: A tutorial. Speech Communication 2004;43:103–21. 10.1016/j.specom.2004.02.004

[53] Baayen RH, Davidson DJ, Bates DM. Mixed-effects modeling with crossed random effects for subjects and items. J Mem Lang 2008;59:390–412. 10.1016/j.jml.2007.12.005

[54] Hamilton MC. Masculine bias in the attribution of personhood: People = Male, Male = People. Psychol Women Q 1991;15:393–402. 10.1111/j.1471-6402.1991.tb00415.x

[55] Audring J. Pronominal gender in spoken Dutch. Journal of Germanic Linguistics 2006;18:85–116. 10.1017/S1470542706000043

[56] Grainger J, Jacobs AM. Masked partial-word priming in visual word recognition: Effects of positional letter frequency 1993;19:951–64. 10.1037//0096-1523.19.5.951 8228845

[57] Marslen-Wilson W, Zwitserlood P. Accessing spoken words: The importance of word onsets. J Exp Psychol Hum Percept Perform 1989;15:576–85. 10.1037/0096-1523.15.3.576

[58] Reali C, Esaulova Y, Öttl A, von Stockhausen L. Role descriptions induce gender mismatch effects in eye movements during reading. Front Psychol 2015;6:1607:1–13. 10.3389/fpsyg.2015.01607 26579003 PMC4630541

[59] Reali C, Esaulova Y, von Stockhausen L. Isolating stereotypical gender in a grammatical gender language: Evidence from eye movements. Appl Psycholinguist 2015;36:977–1006. 10.1017/S0142716414000010

[60] Cacciari C, Padovani R. Further evidence of gender stereotype priming in language: Semantic facilitation and inhibition in Italian role nouns. Appl Psycholinguist 2007;28:277–93. 10.1017/S0142716407070142

[61] Siyanova-Chanturia A, Pesciarelli F, Cacciari C. The electrophysiological underpinnings of processing gender stereotypes in language. PLoS ONE 2012;7 10.1371/journal.pone.0048712 23226494 PMC3513306

[62] Banaji M, Hardin C. Automatic stereotyping. Psychol Sci 1996;7:136–41. 10.1111/j.1467-9280.1996.tb00346.x

[63] McCreary DR. The male role and avoiding femininity. Sex Roles 1994;31:517–31. 10.1007/BF01544277

[64] Feinman S. Why is cross-sex-role behavior more approved for girls than for boys? A status characteristic approach. Sex Roles 1981;7:289–300. 10.1007/BF00287543

[65] Sirin SR, McCreary DR, Mahalik JR. Differential reactions to men and women’s gender role transgressions: Perceptions of social status, sexual orientation, and value dissimilarity. J Mens Stud 2004;12:119–32. 10.3149/jms.1202.119

[66] Zucker KJ, Bradley SJ, Sanikhani M. Sex differences in referral rates of children with gender identity disorder: Some hypotheses. J Abnorm Child Psychol 1997;25:217–27. 10.1023/A:1025748032640 9212374

[67] Van den Brink D, Van Berkum JJ, Bastiaansen MC, Tesink CM, Kos M, Buitelaar JK, et al Empathy matters: ERP evidence for inter-individual differences in social language processing. Soc Cogn Affect Neurosci 2012;7:173–83. 10.1093/scan/nsq094 21148175 PMC3277364

[68] Van Berkum JJA, van den Brink D, Tesink CMJY, Kos M, Hagoort P. The Neural Integration of Speaker and Message. J Cogn Neurosci 2008;20:580–91. 10.1162/jocn.2008.20054 18052777

[69] Camarata S, Woodcock R. Sex differences in processing speed: Developmental effects in males and females. Intelligence 2006;34:231–52. 10.1016/j.intell.2005.12.001

[70] Roivainen E. Gender differences in processing speed: A review of recent research. Learn Individ Differ 2011;21:145–9. 10.1016/j.lindif.2010.11.021

