# Supplementary material for: The processing of the Dutch masculine generic zijn ‘his’ across stereotype contexts: An eye-tracking study
Source: PLoS One. 2018 Oct 18;13(10):e0205903. doi: 10.1371/journal.pone.0205903 (PMC6193704; doi:10.1371/journal.pone.0205903)
Supplement: S1 Appendix — (PDF) [file pone.0205903.s001.pdf]

Experimental stimuli

Nr.	List	Stereotype	Continuation	Item
1	1	female	female	Iedereen was zijn nagels aan het lakken. Zo was ook Amber een felle nagellak aan het aanbrengen.
1	2	female	male	Iedereen was zijn nagels aan het lakken. Zo was ook David een felle nagellak aan het aanbrengen.
2	2	female	female	Iedereen was zijn wimpers aan het verven. Zo was ook Esther mascara aan het opdoen voor donkere wimpers.
2	1	female	male	Iedereen was zijn wimpers aan het verven. Zo was ook Sander mascara aan het opdoen voor donkere wimpers.
3	1	female	female	Iedereen was zijn wenkbrauwen aan het epileren. Zo was ook Nienke met een pincet bezig om de wenkbrauwen een mooie vorm te geven.
3	2	female	male	Iedereen was zijn wenkbrauwen aan het epileren. Zo was ook Wesley met een pincet bezig om de wenkbrauwen een mooie vorm te geven.
4	2	female	female	Iedereen was zijn handtas aan het inpakken. Zo was ook Anna alles aan het pakken om te gaan stappen.
4	1	female	male	Iedereen was zijn handtas aan het inpakken. Zo was ook Adam alles aan het pakken om te gaan stappen.
5	1	female	female	Iedereen was zijn oorbellen aan het indoen. Zo was ook Femke een mooi gouden paar aan het insteken.
5	2	female	male	Iedereen was zijn oorbellen aan het indoen. Zo was ook Ruben een mooi gouden paar aan het insteken.
6	2	female	female	Iedereen was zijn balletschoenen aan het aantrekken. Zo was ook Romy zich aan het klaarmaken voor het optreden.
6	1	female	male	Iedereen was zijn balletschoenen aan het aantrekken. Zo was ook Timo zich aan het klaarmaken voor het optreden.

7	1	female	female	Iedereen was zijn cupcakes aan het versieren. Zo was ook Manon mooie toefjes op de lekkere baksels aan het spuiten.
7	2	female	male	Iedereen was zijn cupcakes aan het versieren. Zo was ook Floris mooie toefjes op de lekkere baksels aan het spuiten.
8	2	female	female	Iedereen was zijn dagcrème aan het aanbrengen. Zo was ook Tessa een likje aan het aanbrengen om een droge huid te voorkomen.
8	1	female	male	Iedereen was zijn dagcrème aan het aanbrengen. Zo was ook Pepijn een likje aan het aanbrengen om een droge huid te voorkomen.
9	1	female	female	Iedereen was zijn roddelblad aan het lezen. Zo was ook Laura helemaal verdiept in de verhalen over verschillende beroemdheden.
9	2	female	male	Iedereen was zijn roddelblad aan het lezen. Zo was ook Joris helemaal verdiept in de verhalen over verschillende beroemdheden.
10	2	female	female	Iedereen was zijn haar aan het föhnen. Zo was ook Sterre het haar aan het drogen in het zwembad.
10	1	female	male	Iedereen was zijn haar aan het föhnen. Zo was ook Wouter het haar aan het drogen in het zwembad.
11	1	female	female	Iedereen was zijn paard aan het borstelen. Zo was ook Sarah de vacht aan het schoonmaken na een lange rit.
11	2	female	male	Iedereen was zijn paard aan het borstelen. Zo was ook Justin de vacht aan het schoonmaken na een lange rit.
12	2	female	female	Iedereen was zijn hart aan het uitstorten. Zo was ook Fenna over de problemen op het werk aan het vertellen.
12	1	female	male	Iedereen was zijn hart aan het uitstorten. Zo was ook Kevin over de problemen op het werk aan het vertellen.
13	1	female	female	Iedereen was zijn dagboek aan het bijhouden. Zo was ook Sanne aan het opschrijven wat er deze dag te beleven was geweest.

13	2	female	male	Iedereen was zijn dagboek aan het bijhouden. Zo was ook Jeroen aan het opschrijven wat er deze dag te beleven was geweest.
14	2	female	female	Iedereen was zijn yogaoefeningen aan het doen. Zo was ook Lieke goed bezig met een oefening.
14	1	female	male	Iedereen was zijn yogaoefeningen aan het doen. Zo was ook Peter goed bezig met een oefening.
15	1	female	female	Iedereen was zijn prosecco aan het drinken. Zo was ook Esmee een flinke slok aan het nemen.
15	2	female	male	Iedereen was zijn prosecco aan het drinken. Zo was ook Jesse een flinke slok aan het nemen.
16	2	female	female	Iedereen was zijn kaarsen aan het aansteken. Zo was ook Kelly wat lichtjes aan het branden om een leuke sfeer te creëren.
16	1	female	male	Iedereen was zijn kaarsen aan het aansteken. Zo was ook Lucas wat lichtjes aan het branden om een leuke sfeer te creëren.
17	1	neutral	female	Iedereen was zijn vakantie aan het plannen. Zo was ook Judith op zoek naar mooie bestemmingen.
17	2	neutral	male	Iedereen was zijn vakantie aan het plannen. Zo was ook Jelle op zoek naar mooie bestemmingen.
18	2	neutral	female	Iedereen was zijn presentatie aan het voorbereiden. Zo was ook Bente nog met de opmaak van de slides bezig.
18	1	neutral	male	Iedereen was zijn presentatie aan het voorbereiden. Zo was ook Jacob nog met de opmaak van de slides bezig.
19	1	neutral	female	Iedereen was zijn boodschappen aan het doen. Zo was ook Myrthe nog wat ingrediënten voor het avondeten aan het kopen.
19	2	neutral	male	Iedereen was zijn boodschappen aan het doen. Zo was ook Thomas nog wat ingrediënten voor het avondeten aan het kopen.
20	2	neutral	female	Iedereen was zijn ouders aan het bezoeken. Zo was ook Mandy weer een keer thuis bij de ouders.
20	1	neutral	male	Iedereen was zijn ouders aan het bezoeken. Zo was ook Dennis weer een keer thuis bij de ouders.

21	1	neutral	female	Iedereen was zijn telefoon aan het checken. Zo was ook Merel tijdens het college de hele tijd berichtjes aan het beantwoorden.
21	2	neutral	male	Iedereen was zijn telefoon aan het checken. Zo was ook Jasper tijdens het college de hele tijd berichtjes aan het beantwoorden.
22	2	neutral	female	Iedereen was zijn fiets aan het stallen. Zo was ook Lotte de fiets bij het station aan het neerzetten.
22	1	neutral	male	Iedereen was zijn fiets aan het stallen. Zo was ook Marco de fiets bij het station aan het neerzetten.
23	1	neutral	female	Iedereen was zijn tanden aan het poetsen. Zo was ook Daphne zich aan het klaarmaken om naar bed te gaan.
23	2	neutral	male	Iedereen was zijn tanden aan het poetsen. Zo was ook Robert zich aan het klaarmaken om naar bed te gaan.
24	2	neutral	female	Iedereen was zijn lunch aan het eten. Zo was ook Marit een boterham met kaas aan het nuttigen.
24	1	neutral	male	Iedereen was zijn lunch aan het eten. Zo was ook Gerrit een boterham met kaas aan het nuttigen.
25	1	neutral	female	Iedereen was zijn veters aan het strikken. Zo was ook Maaike zich aan het klaarmaken om naar buiten te gaan.
25	2	neutral	male	Iedereen was zijn veters aan het strikken. Zo was ook Stefan zich aan het klaarmaken om naar buiten te gaan.
26	2	neutral	female	Iedereen was zijn OV-kaart aan het opladen. Zo was ook Linda geld erop aan het zetten op het station.
26	1	neutral	male	Iedereen was zijn OV-kaart aan het opladen. Zo was ook Remco geld erop aan het zetten op het station.
27	1	neutral	female	Iedereen was zijn paper aan het schrijven. Zo was ook Inge druk bezig om de deadline te halen.
27	2	neutral	male	Iedereen was zijn paper aan het schrijven. Zo was ook Tygo druk bezig om de deadline te halen.

28	2	neutral	female	Iedereen was zijn ramen aan het opendoen. Zo was ook Anouk wat frisse lucht aan het binnenlaten.
28	1	neutral	male	Iedereen was zijn ramen aan het opendoen. Zo was ook Dylan wat frisse lucht aan het binnenlaten.
29	1	neutral	female	Iedereen was zijn koffie aan het opdrinken. Zo was ook Sophie aan het proberen het kopje voor de les leeg te krijgen.
29	2	neutral	male	Iedereen was zijn koffie aan het opdrinken. Zo was ook Willem aan het proberen het kopje voor de les leeg te krijgen.
30	2	neutral	female	Iedereen was zijn hond aan het uitlaten. Zo was ook Emma met de trouwe viervoeter naar het park gegaan.
30	1	neutral	male	Iedereen was zijn hond aan het uitlaten. Zo was ook Hugo met de trouwe viervoeter naar het park gegaan.
31	1	neutral	female	Iedereen was zijn gitaar aan het stemmen. Zo was ook Esther aan de stemmechanieken aan het draaien.
31	2	neutral	male	Iedereen was zijn gitaar aan het stemmen. Zo was ook Sander aan de stemmechanieken aan het draaien.
32	2	neutral	female	Iedereen was zijn afval aan het wegbrengen. Zo was ook Nienke het vuilnis aan het buitenzetten.
32	1	neutral	male	Iedereen was zijn afval aan het wegbrengen. Zo was ook Wesley het vuilnis aan het buitenzetten.
33	1	male	female	Iedereen was zijn aandelen aan het verkopen. Zo was ook Anna aan het proberen om meer verlies te voorkomen.
33	2	male	male	Iedereen was zijn aandelen aan het verkopen. Zo was ook Adam aan het proberen om meer verlies te voorkomen.
34	2	male	female	Iedereen was zijn computer aan het repareren. Zo was ook Femke een nieuwe harde schijf aan het inbouwen.
34	1	male	male	Iedereen was zijn computer aan het repareren. Zo was ook Ruben een nieuwe harde schijf aan het inbouwen.

35	1	male	female	Iedereen was zijn mountainbike aan het inladen. Zo was ook Romy het rijwiel achter in het busje aan het plaatsen.
35	2	male	male	Iedereen was zijn mountainbike aan het inladen. Zo was ook Timo het rijwiel achter in het busje aan het plaatsen.
36	2	male	female	Iedereen was zijn golfclubs aan het poetsen. Zo was ook Manon nog wat opgedroogde aarde van de clubs aan het afborstelen.
36	1	male	male	Iedereen was zijn golfclubs aan het poetsen. Zo was ook Floris nog wat opgedroogde aarde van de clubs aan het afborstelen.
37	1	male	female	Iedereen was zijn biceps aan het trainen. Zo was ook Tessa fanatiek bezig met de gewichten.
37	2	male	male	Iedereen was zijn biceps aan het trainen. Zo was ook Pepijn fanatiek bezig met de gewichten.
38	2	male	female	Iedereen was zijn voetbaltrucs aan het oefenen. Zo was ook Laura al urenlang met de bal bezig.
38	1	male	male	Iedereen was zijn voetbaltrucs aan het oefenen. Zo was ook Joris al urenlang met de bal bezig.
39	1	male	female	Iedereen was zijn barbecue aan het aansteken. Zo was ook Sterre de houtskool aan het aanmaken om lekkere steaks te kunnen gaan grillen.
39	2	male	male	Iedereen was zijn barbecue aan het aansteken. Zo was ook Wouter de houtskool aan het aanmaken om lekkere steaks te kunnen gaan grillen.
40	2	male	female	Iedereen was zijn auto aan het waxen. Zo was ook Sarah bezig om de auto weer te laten glimmen.
40	1	male	male	Iedereen was zijn auto aan het waxen. Zo was ook Justin bezig om de auto weer te laten glimmen.
41	1	male	female	Iedereen was zijn sigaar aan het roken. Zo was ook Fenna trekjes aan het nemen van een havanna.
41	2	male	male	Iedereen was zijn sigaar aan het roken. Zo was ook Kevin trekjes aan het nemen van een havanna.
42	2	male	female	Iedereen was zijn vrachtwagen aan het parkeren. Zo was ook Sanne de vrachtwagen bij een tankstation aan het neerzetten voor een pauze.

42	1	male	male	Iedereen was zijn vrachtwagen aan het parkeren. Zo was ook Jeroen de vrachtwagen bij een tankstation aan het neerzetten voor een pauze.
43	1	male	female	Iedereen was zijn autobanden aan het verwisselen. Zo was ook Lieke weer de zomerbanden onder de auto aan het zetten.
43	2	male	male	Iedereen was zijn autobanden aan het verwisselen. Zo was ook Peter weer de zomerbanden onder de auto aan het zetten.
44	2	male	female	Iedereen was zijn snor aan het scheren. Zo was ook Esmee met het scheerapparaat in de weer.
44	1	male	male	Iedereen was zijn snor aan het scheren. Zo was ook Jesse met het scheerapparaat in de weer.
45	1	male	female	Iedereen was zijn whisky aan het drinken. Zo was ook Kelly aan het genieten van een glaasje single malt.
45	2	male	male	Iedereen was zijn whisky aan het drinken. Zo was ook Lucas aan het genieten van een glaasje single malt.
46	2	male	female	Iedereen was zijn haargel aan het indoen. Zo was ook Judith het haar aan het stylen met wat gel.
46	1	male	male	Iedereen was zijn haargel aan het indoen. Zo was ook Jelle het haar aan het stylen met wat gel.
47	1	male	female	Iedereen was zijn pijp aan het roken. Zo was ook Bente Deense natuurtabak aan het paffen.
47	2	male	male	Iedereen was zijn pijp aan het roken. Zo was ook Jacob Deense natuurtabak aan het paffen.
48	2	male	female	Iedereen was zijn land aan het ploegen. Zo was ook Myrthe een akker aan het voorbereiden voor nieuw zaad.
48	1	male	male	Iedereen was zijn land aan het ploegen. Zo was ook Thomas een akker aan het voorbereiden voor nieuw zaad.

Controls

Nr.	List	Stereotype	Continuation	Item
1	1	neutral	female	Iedereen was broodjes aan het smeren. Zo was ook Mandy wat pistoletjes aan het beleggen met ham en kaas.

1	2	neutral	male	Iedereen was broodjes aan het smeren. Zo was ook Dennis wat pistoletjes aan het beleggen met ham en kaas.
2	2	neutral	female	Iedereen was de post aan het lezen. Zo was ook Merel een brief van de belastingdienst aan het doornemen.
2	1	neutral	male	Iedereen was de post aan het lezen. Zo was ook Jasper een brief van de belastingdienst aan het doornemen.
3	1	neutral	female	Iedereen was geld aan het uitgeven. Zo was ook Lotte allerlei nieuwe spullen aan het kopen.
3	2	neutral	male	Iedereen was geld aan het uitgeven. Zo was ook Marco allerlei nieuwe spullen aan het kopen.
4	2	neutral	female	Iedereen was de huur aan het overmaken. Zo was ook Daphne het bedrag naar de huisbaas aan het overschrijven.
4	1	neutral	male	Iedereen was de huur aan het overmaken. Zo was ook Robert het bedrag naar de huisbaas aan het overschrijven.
5	1	neutral	female	Iedereen was ballonnen aan het opblazen. Zo was ook Marit met de voorbereidingen voor het feest aan het helpen.
5	2	neutral	male	Iedereen was ballonnen aan het opblazen. Zo was ook Gerrit met de voorbereidingen voor het feest aan het helpen.
6	2	neutral	female	Iedereen was de baas aan het bellen. Zo was ook Maaike aan het proberen om de baas aan de lijn te krijgen.
6	1	neutral	male	Iedereen was de baas aan het bellen. Zo was ook Stefan aan het proberen om de baas aan de lijn te krijgen.
7	1	neutral	female	Iedereen was ski’s aan het aandoen. Zo was ook Linda zich aan het klaarmaken voor de piste.
7	2	neutral	male	Iedereen was ski’s aan het aandoen. Zo was ook Remco zich aan het klaarmaken voor de piste.
8	2	neutral	female	Iedereen was een paraplu aan het opendoen. Zo was ook Inge zich aan het beschermen tegen de regen.
8	1	neutral	male	Iedereen was een paraplu aan het opendoen. Zo was ook Tygo zich aan het beschermen tegen de regen.

9	1	neutral	female	Iedereen was cadeautjes aan het uitpakken. Zo was ook Anouk met veel plezier inpakpapier van een groot cadeau aan het afhalen.
9	2	neutral	male	Iedereen was cadeautjes aan het uitpakken. Zo was ook Dylan met veel plezier inpakpapier van een groot cadeau aan het afhalen.
10	2	neutral	female	Iedereen was de krant aan het lezen. Zo was ook Sophie zich op de hoogte aan het stellen van het wereldnieuws.
10	1	neutral	male	Iedereen was de krant aan het lezen. Zo was ook Willem zich op de hoogte aan het stellen van het wereldnieuws.
11	1	neutral	female	Iedereen was de cijfers aan het bekijken. Zo was ook Emma de resultaten van het tentamen aan het bestuderen.
11	2	neutral	male	Iedereen was de cijfers aan het bekijken. Zo was ook Hugo de resultaten van het tentamen aan het bestuderen.
12	2	neutral	female	Iedereen was een treinkaartje aan het kopen. Zo was ook Amber bij het loket in de rij gaan staan.
12	1	neutral	male	Iedereen was een treinkaartje aan het kopen. Zo was ook David bij het loket in de rij gaan staan.
