# Supplementary figures and images for: The processing of the Dutch masculine generic zijn ‘his’ across stereotype contexts: An eye-tracking study
Source: PLoS One. 2018 Oct 18;13(10):e0205903. doi: 10.1371/journal.pone.0205903 (PMC6193704; doi:10.1371/journal.pone.0205903)

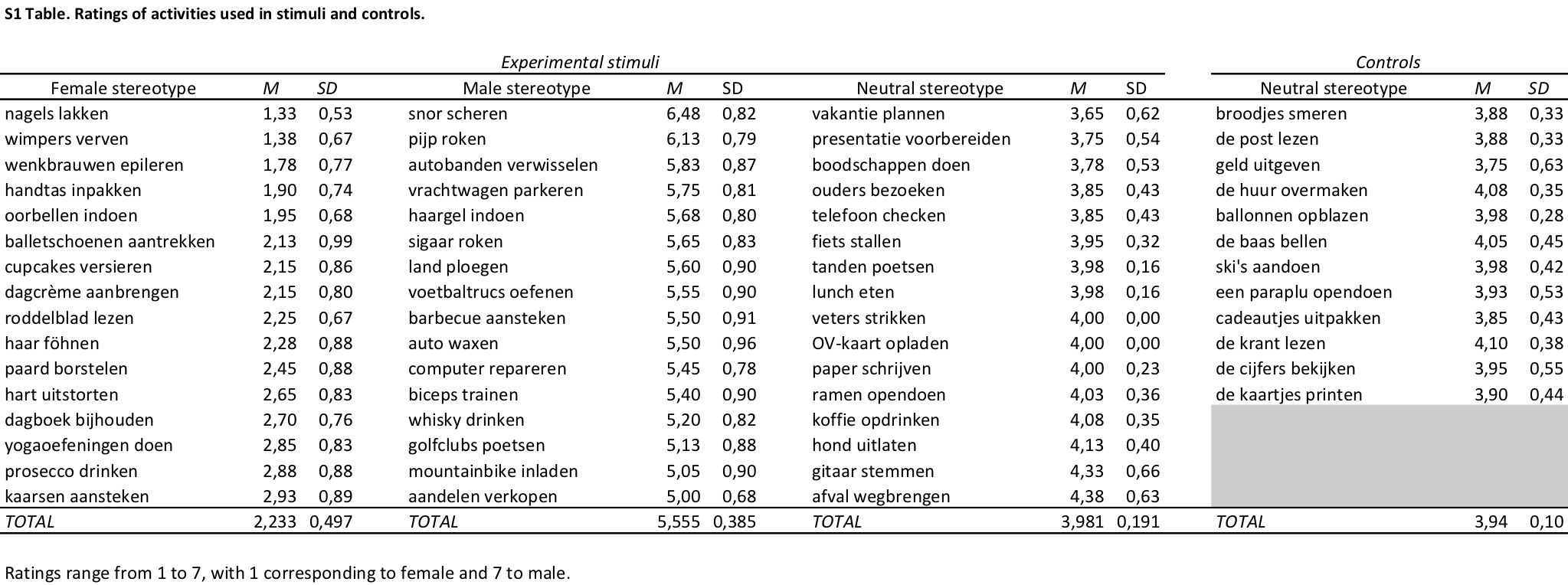

Supplement: S1 Table — Ratings range from 1 to 7, with 1 corresponding to female and 7 to male. (PNG) [file pone.0205903.s002.png]

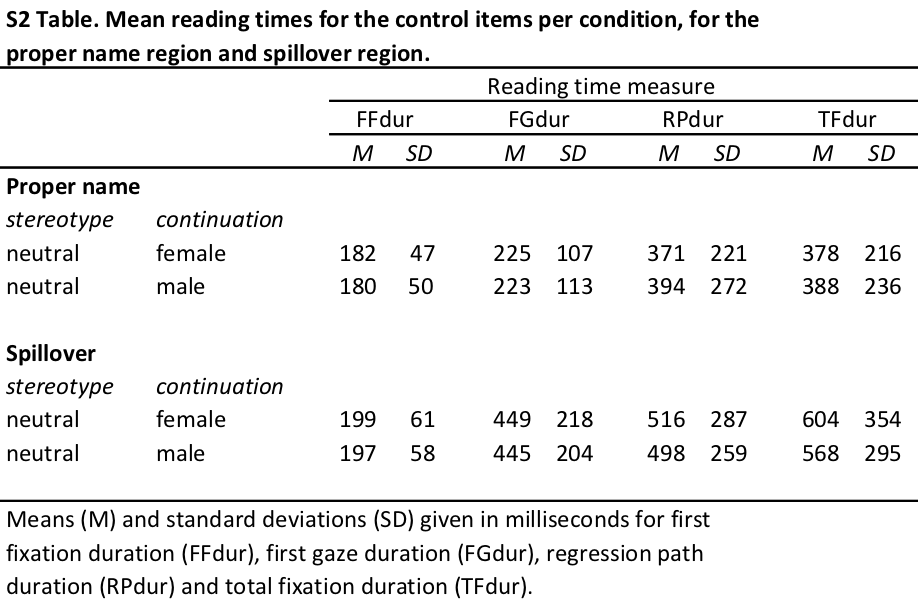

Supplement: S2 Table — Means (M) and standard deviations (SD) given in milliseconds for first fixation duration (FFdur), first gaze duration (FGdur), regression path duration (RPdur) and total fixation duration (TFdur). (PNG) [file pone.0205903.s003.png]

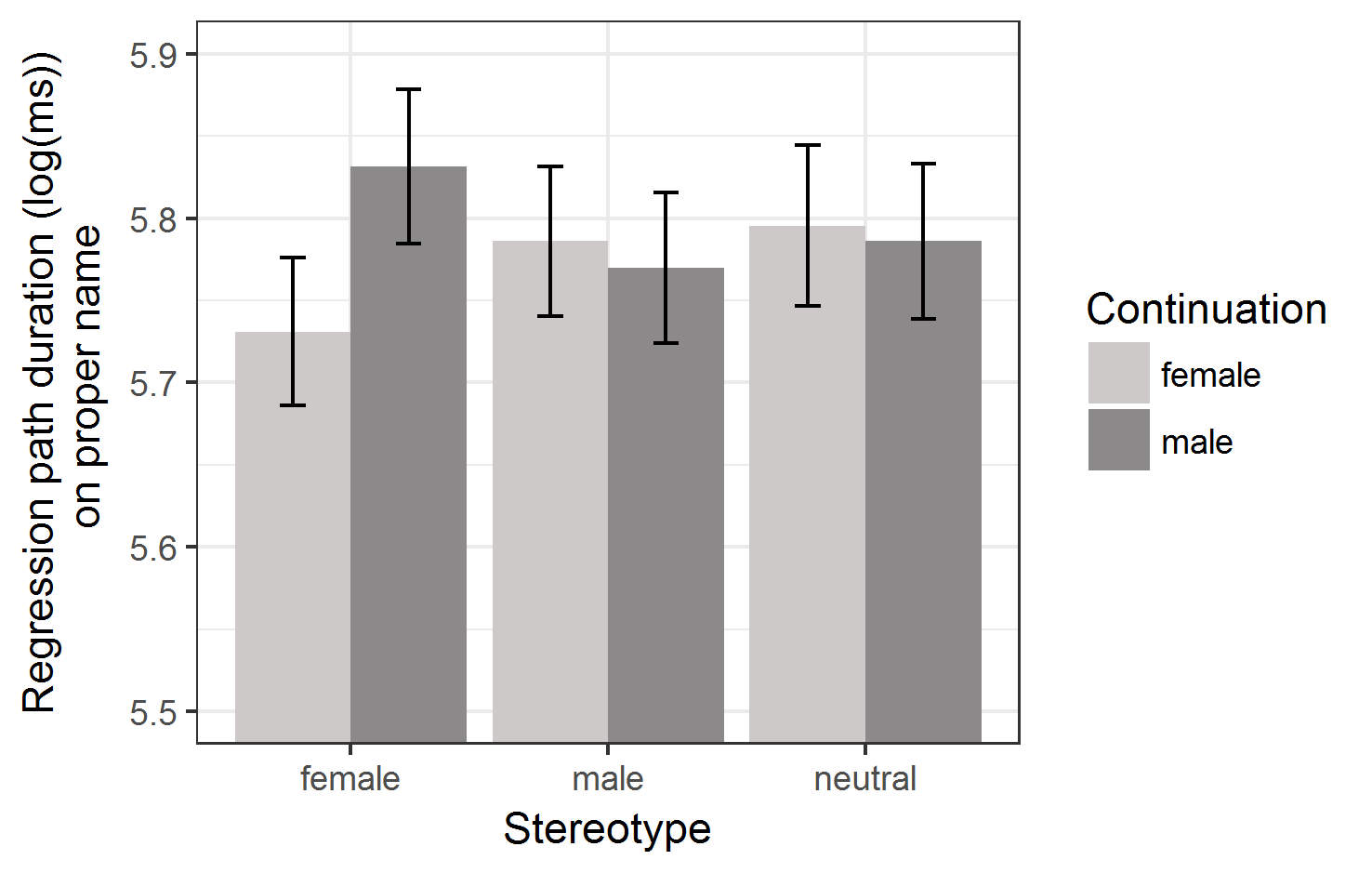

Supplement: S1 Fig — Condition means are given in log milliseconds. Error bars represent 95% confidence intervals. (TIF) [file pone.0205903.s004.tif]

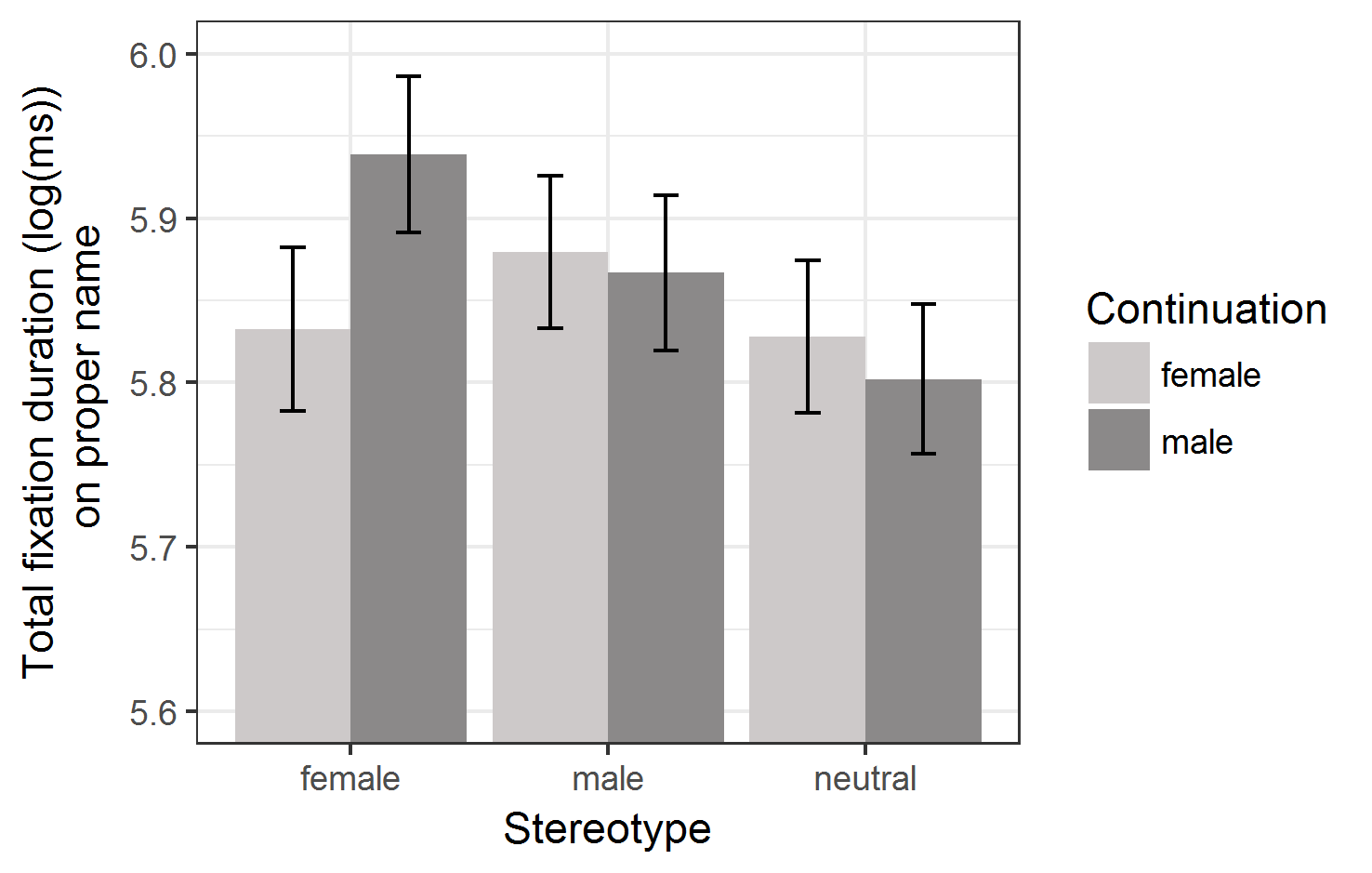

Supplement: S2 Fig — Condition means are given in log milliseconds. Error bars represent 95% confidence intervals. (TIF) [file pone.0205903.s005.tif]

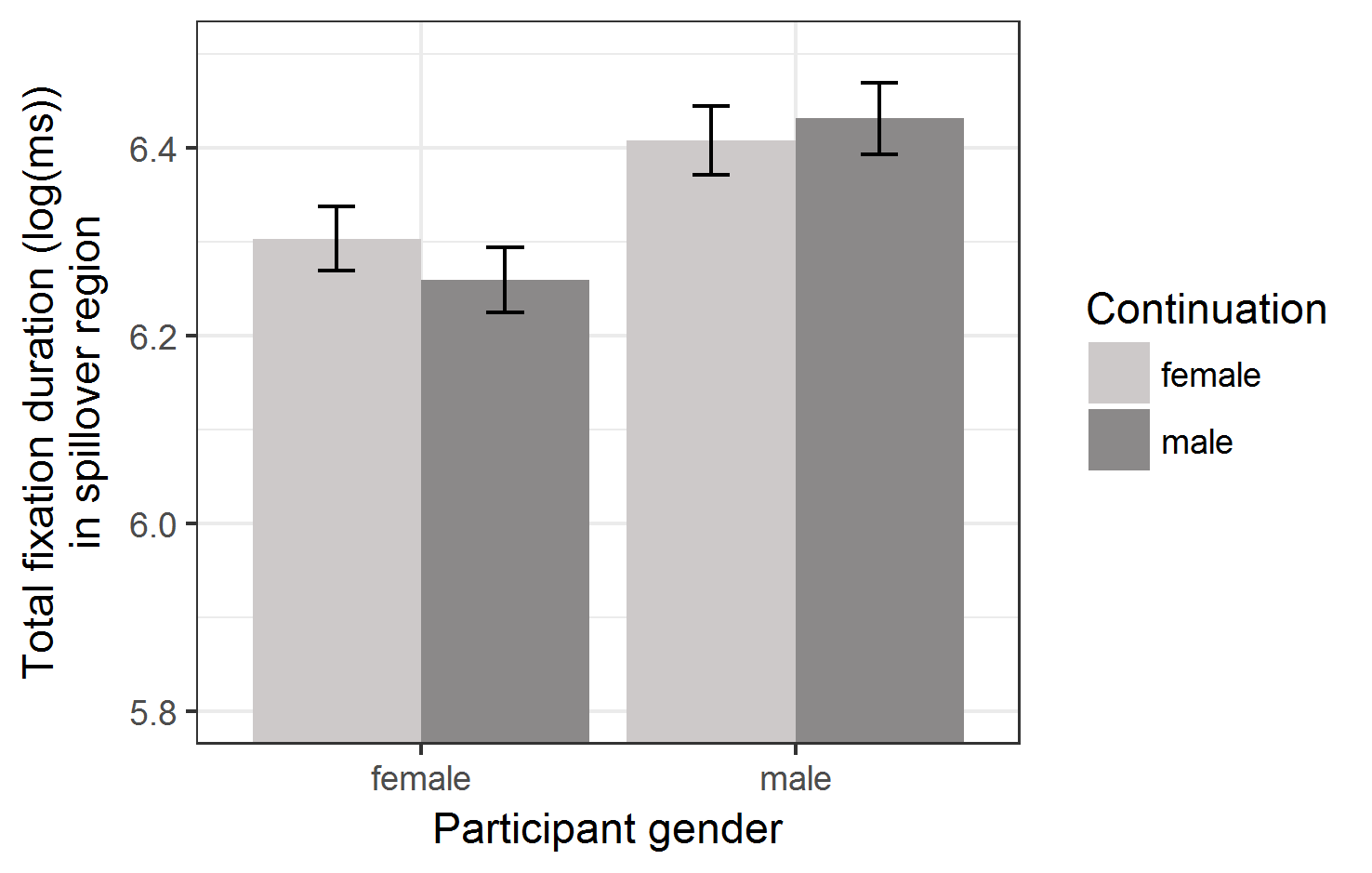

Supplement: S3 Fig — Condition means are given in log milliseconds. Error bars represent 95% confidence intervals. (TIF) [file pone.0205903.s006.tif]
